# Transcriptome and metabolite profiling reveals the mechanism of hepatic lipid metabolism during fasting in chicken

**DOI:** 10.5713/ab.25.0014

**Published:** 2025-08-12

**Authors:** Lu Xu, Zhe Liu, Mengling Gong, Zhiheng Wei, Yang Gu, Lisha Yu, Jianfeng Yu, Zhiliang Gu

**Affiliations:** 1 School of Biology and Food Engineering, Changshu Institute of Technology, Changshu, China; 2 Medical Engineering Department, Cancer Affiliated Hospital of Xinjiang Medical University, Urumqi, China

**Keywords:** Chicken, *EHHADH* Gene, Fasting, Lipid Metabolism, Liver

## Abstract

**Objective:**

Since the liver is key to poultry lipid metabolism and fasting models are widely used in studying animal nutrition metabolism, this study used fasting to explore chicken liver lipid metabolism characteristics, providing a basis for poultry lipid metabolism research.

**Methods:**

RNA-seq and metabolomics were combined to analyze 72-hour fasting effects on chicken liver lipid metabolism. Key lipid metabolism-related genes and metabolites were identified, with their mechanisms explored via RNAi and Oil Red O staining.

**Results:**

Metabolomics identified 648 differential metabolites, including 8 (e.g., Arachidonoyl amine) with levels 160-fold higher than controls. Transcriptomics found 849 differentially expressed genes (DEGs), 78 involved in lipid metabolism; Protein–protein interaction analysis revealed hub genes like *EHHADH*. Transcriptome-metabolome correlation analysis showed 101 DEGs correlated with 99 metabolites, with *EHHADH* associated with 54 metabolites (the most) and linked to 2-methylcrotonoyl-CoA and 5 pathways via KEGG Markup Language. Fasting upregulated *EHHADH*, whose overexpression/interference altered mRNA levels of *Fabp7*, *Plin2*, *ACACA*, *FASN*, *PPARα*, as well as cholesterol/triglyceride levels. *EHHADH* overexpression reduced LMH cell lipid deposition, while interference increased it, indicating its role in reducing lipid deposition.

**Conclusion:**

Fasting significantly alters chicken liver lipid metabolism, and *EHHADH* helps reduce liver lipid deposition.

## INTRODUCTION

The liver is the second largest fat storage organ after adipose tissue in poultry [1]. Abnormal fatty acid oxidation, lipoprotein transport, or fat synthesis will cause excessive lipid accumulation of fat in the liver, thereby inducing excessive lipid deposition [2]. Excessive lipid deposition is one of the factors affecting the low feed conversion rate in poultry.

Energy is the primary need of animals, providing support for all their activities. The energy/nutritional level of animals is closely related to the performance of their production. The nutritional and energy demands of the body vary with different growth stages. Commonly used animal nutrition research models include fasting/re-feeding models, overfeeding models, high-sugar and high-fat feeding models, and restricted feeding compensatory growth models. Animal models with nutritional deficiency or insufficiency can not only simulate the effects of low nutrition/energy levels on animal growth and development and production performance, but also simulate the effects of low nutrition levels on immune response and health. Song et al [3] reported that 48 hours of fasting reduced the fat synthesis capacity in chick livers and increased the ratios of phosphorylated AMPKα to total AMPKα and phosphorylated liver kinase B1 (LKB1) to total LKB1. Fasting activated the LKB1/AMPK signaling pathway. After refeeding for 24 hours, the above ratios decreased to the contents of ad libitum feeding, suggesting that refeeding reversed the activation of AMPK induced by fasting, and the LKB1/AMPK signaling pathway was involved in the regulation of energy homeostasis in chickens during fasting. Fasting decreased the expression of key fat synthesis-related genes, such as stearoyl-CoA desaturase (*SCD*), glucocorticoid receptor (*GR*) and fatty acid synthase (*FAS*), leading to decreased glucose utilization and cholesterol and fatty acid synthesis in chicken liver [4].

In the current study, we fasted broilers for 72 hours and analyzed the effect of fasting on transcriptome and metabolomics in the chicken liver. We also correlated the transcriptome to metabolomics data to detect the relationship between the hub genes and metabolites. And also, RNAi and oil red staining were used to further analyze the function of the hub gene.

## MATERIALS AND METHODS

### Animals and sample collection

Sixty healthy, 2-week-old yellow-feather broilers were purchased from the Xinfeng Village Chicken Farm. All chickens were kept at 25°C in separate cages (35×38×42 cm^3^). The chickens were fed a standard broiler diet three times daily before the start of the trial. A total of 60 chickens were divided into control and fasting groups with 30 chickens per group. The chickens in the control group were fed a standard diet for broilers, whereas the chickens in the fasting group were fasted for 72 hours. During this 72-hour period, chickens in both groups had free access to water. Whole blood samples from the control and experimental groups were collected using the wing vein blood sampling method, placed on ice for 10 mins, centrifuged at 251×g for 15 mins, and the serum was collected. Subsequently, the intact livers of the two groups were isolated, snap-frozen in liquid nitrogen, and stored at −80°C until later use.

### Serum triglyceride, total cholesterol, low density lipoprotein-C, and glucose assays

Four biochemical index assay kits were purchased from Nanjing Jiancheng Bioengineering Institute. The kit numbers were A113-1 (low density lipoprotein-C [LDL-C]), A110-1 (triglycerides), A154-1-1 (glucose), and A111-1-1 (total cholesterol). The reaction solution was prepared according to the manufacturer’s instructions for the three indicators of total cholesterol, triglycerides, and glucose in serum and incubated 37°C for 10 mins. The detection was performed using a microplate reader (Thermo Fisher Scientific). Triglycerides were measured at 510 nm, total cholesterol at 500 nm, and glucose at 505 nm. Sample concentrations were calculated:


(1) $$\frac{\left({\text{A}}_{\text{test}}-{\text{A}}_{\text{control}}\right)}{\left({\text{A}}_{\text{standard}}-{\text{A}}_{\text{control}}\right)}\times {\text{C}}_{\text{standard}}\times \text{Sample dilution factor}$$

Where for glucose, C_standard_ = 5.55 mmol/L; for total cholesterol, C_standard_ = 5.17 mmol/L; and for triglycerides, C_standard_ = 2.26 mmol/L.

Serum LDL-C was determined. The first reaction solution was prepared according to the manufacturer’s instructions, incubated at 37°C for 5 mins, and the absorbance value was measured at 600 nm using a microplate reader and recorded as A1. The second reaction solution was added, mixed, and incubated at 37°C for 5 mins. The second detection was performed under the same conditions and denoted as A2. ΔA was calculated as the difference between A1 and A2. The amount of LDL-C in the serum was calculated according to the following formula:


(2) $$\text{Concentration of LDL-C}=\frac{\left(\Delta {\text{A}}_{\text{test}}-\Delta {\text{A}}_{\text{control}}\right)}{\left(\Delta {\text{A}}_{\text{standard}}-\Delta {\text{A}}_{\text{control}}\right)}\times {\text{C}}_{\text{standard}}$$

Where C_standard_ = 2.55 mmol/L. One-way analysis of variance (ANOVA) was used for statistical analysis of the results.

### RNA extraction and quantitative reverse transcription polymerase chain reaction

Total RNA was isolated using an RNAprep Pure Tissue Kit (Tiangen Biotech). Select five individuals from each group, weigh 100 mg of liver tissue from each individual to extract total RNA for the RNA-seq experiment. In addition, select three individuals from each group, and weigh 50 mg of liver from each individual to extract total RNA for the quantitative experiment. Extract the total RNA from the liver tissues of the fasting group and the control group according to the instructions of the kit.

The quality of RNA-seq samples was assessed using the Agilent 2100 Bioanalyzer (Agilent Technologies). The quality of quantification samples was tested via Nanodrop (Thermo Fisher Scientific)

Reverse transcription was performed using a PrimeScript RT reagent kit (Perfect Real Time) (Takara Bio). The work solution was: 5× PrimeScript buffer (for Real Time), 2 μL; PrimeScript RT enzyme mix I, 0.5 μL; Oligo dT primer (50 μM), 0.5 μL; Random 6-mers (100 μM), 0.5 μL; total RNA, 5 μL; and RNase-free dH_2_O, 1.5 μL. The mixture was incubated at 37°C for 15 min and then treated at 85°C for 5 s.

Reverse transcription quantitative polymerase chain reaction (RT-qPCR) was performed using a TB Green Advantage qPCR premix (Takara Bio), according to the manufacturer’s instructions. The primers used for qPCR are listed in Table 1. The *beta-actin* gene was used as a reference gene. The 2^−ΔΔCT^ method was used for calculating the relative expression of mRNAs. ANOVA was used for statistical analysis of the results.

### RNA sequencing and sequence library construction

Approximately 5 μg of total RNA was used for sequencing. Total RNA was digested with DNase I and mRNAs were enriched with oligo (dT) magnetic beads. The mRNAs were fragmented and used as templates to synthesize first-strand cDNA using random hexamers. Second-strand cDNA was then synthesized by adding buffer, dNTPs, RNase H, and DNA polymerase I. Subsequently, cDNA was purified using a QIAquick PCR purification kit (Qiagen). Next, base A was added to the 3′ end of cDNA using Klenow Exo (New England Biolabs), and the sequencing adapters were ligated to the ends of cDNA using T4 DNA ligase (Takara Bio). The ligated cDNA fragments were separated using gel electrophoresis, followed by gel purification using a QIAquick gel extraction kit (Qiagen). Purified cDNA fragments were amplified using PCR. The quality of the constructed library was determined using the Agilent 2100 Bioanalyzer (Agilent Technologies). RNA-seq libraries were paired-end sequenced using an Illumina HiSeq 2500 (Illumina).

### Bioinformatics analysis of RNA-seq data

Raw sequencing data were processed to obtain clean reads by removing adaptor-ligated contaminants, low-quality reads, and short-read tags using Trimmomatic [5] software. HISAT [6] was used to map clean reads to the reference chicken genome (genome assembly: bGalGal1.mat.broiler.GRCg7b). Gene expression levels were calculated as fragments per kilobase of transcript per million mapped reads (FPKM) [7]. Differentially expressed genes (DEGs) were analyzed using DESeq software (REF). Genes with |log_2_(fold change)|>1 and p<0.05 were identified as DEGs. Protein–protein interaction (PPI) analysis of DEGs was performed using Cytoscape 3.10.1 (REF). Gene Ontology (GO) and Kyoto Encyclopedia of Genes and Genomes (KEGG) pathway analyses were performed using DAVID (https://davidbioinformatics.nih.gov/) and KOBAS (http://bioinfo.org/kobas) programs, respectively.

### Sample preparation for liquid chromatography–mass spectrometry

Liver samples for metabolomic assays were prepared. Thirty milligrams of liver was mixed with 20 μL of internal standard L-2-chlorophenylalanine (0.3 mg/mL), 20 μL of Lyso PC17:0 (0.01 mg/mL), both in methanol configuration, and 400 μL aqueous methanol solution (CH_3_OH:H_2_O V:V = 4:1). After pre-cooling, the samples were ground at 60 Hz for 2 mins. The samples were subjected to ultrasonic extraction in an ice water bath for 10 mins, and then kept at −20°C for 20 mins. The samples were centrifuged at 18,700×g for 10 mins at 4°C and 300 μL of the supernatant was air-dried and then redissolved in 400 μL of methanol-water mixture (V:V = 1:4) by vortexing for 30 s and sonicating for 2 mins. The samples were then centrifuged at 18,700×g for 10 mins at 4°C. Using a syringe, 150 μL of the supernatant was aspirated, filtered using a 0.22-μm organic phase pinhole filter, transferred to LC injection vials, and stored at −80°C until liquid chromatography–mass spectrometry (LC–MS) analysis. Samples from six chickens were analyzed using LC–MS. Quality control (QC) samples were prepared by mixing equal amounts of each sample.

### Liquid chromatography–mass spectrometry analysis

LC–MS was performed using a system consisting of an AB Exion LC (AB Sciex) and AB TripleTOF 6600 Plus (AB Sciex). The chromatographic column was an Acquity UPLC HSS T3 (100×2.1 mm, 1.8 μm, Waters); column temperature was 45°C. The mobile phase consisted of water (0.1% formic acid; A) and B-acetonitrile (0.1% formic acid). Flow rate was 0.35 mL/min and the injection volume was 2 μL.

The raw LC–MS data were processed using Progenesis QI v2.3 software (Nonlinear Dynamics), including baseline filtering, peak identification, integration, retention time correction, peak alignment, and normalization. The main parameters included the precursor tolerance (5 ppm), product tolerance (10 ppm), and product ion threshold (5%). The metabolites were identified using the metabolome databases HMDB (http://www.hmdb.ca/), Lipidmaps (http://www.lipidmaps.org/), and METLIN (https://metlin.scripps.edu/).

Orthogonal partial least squares–discriminant analysis (OPLS–DA) was performed to determine the metabolic changes in each group. From the OPLS–DA model, the variable importance of projection (VIP) was obtained, in which VIP>1 was used to identify potential metabolites. To prevent model overfitting, seven-fold cross validation and 200 response permutation testing were used to evaluate the quality of the model. Univariate analysis, which included Student’s t-test and fold-change analysis, was used to acquire preliminary differential metabolites. Differential metabolites were identified using the criteria VIP>1 and p-value<0.05. Pathway enrichment analysis of the differential metabolites was performed using the KEGG pathway database (https://www.kegg.jp/).

### Combined analysis of transcriptome and metabolome data

Correlations between DEGs and differential metabolites were analyzed using Pearson’s correlation analysis. The KEGG Markup Language (KGML) was used to analyze the relationship between key DEGs and their corresponding metabolites and signaling pathways to explore the candidate genes involved in chicken liver lipid metabolism. Finally, Venn analysis was used to analyze the signaling pathways of the transcriptome and metabolome and investigate the common or different signaling pathways between them.

### Cell culture and knockdown of the *EHHADH* gene

Chicken-liver-derived LMH cells were purchased from ATCC. Cells were cultured in Waymouth’s medium (Pricella) containing 10% fetal bovine serum (Sangon Biotech) and 100 U/mL penicillin/streptomycin in a humidified incubator at 37°C with 5% CO_2_ in 25-cm^2^ cell culture flask (Sparkjade Scientific Instruments). A total of three siRNAs targeting the *EHHADH* gene (GenePharma), namely siRNA-1011-*EHHADH*, siRNA-1831-*EHHADH*, and siRNA-450-*EHHADH*, were designed and synthesized. Seed cells at a density of 2.5×10^5^ cells into a 24-well plate (Sparkjade Scientific Instruments), culture them in an antibiotic-free medium, and perform transfection 12 hours after seeding. Transfection of siRNA (twenty nanomolar of each siRNA was used for transfection) into chicken LMH cells was performed using the Lipofectamine RNAiMAX transfection reagent (Thermo Fisher Scientific). Total RNA was extracted from cells 48 hours after transfection. Knockdown efficiency was determined using RT-qPCR. ANOVA was used for statistical analysis of the results.

### Construction of *EHHADH* gene overexpression vector and cell transfection

Using the liver cDNA of adult chickens as a template, the CDS fragment of the *EHHADH* gene was amplified by PCR. The primers used were listed in Table 1. The PCR reaction system was as follows: 10×PCR Buffer 2.5 μL, dNTP 2 μL, forward primer 1 μL, reverse primer 1 μL, cDNA 1 μL, rTaq 0.3 μL and ddH_2_O 17.2 μL. The PCR reaction temperatures were as follows: 94°C 5 mins, 94°C 30 s, 63.6°C 30 s, 72°C 130 s, 72°C 10 mins, and keep in 10°C. The reaction was performed for 35 cycles.

The amplified fragment was ligated into the pCMV-3-tag-9 vector. The recombinant plasmid was extracted according to the manufacturer’s instructions for the plasmid extraction kit (DP117) (TIANGEN). Seed cells at a density of 2.5×10^5^ cells into a 24-well plate, culture them in an antibiotic-free medium, and perform transfection 12 hours after seeding. The recombinant plasmid was transfected into LMH cells using Lipomaster 2000 Transfection Reagent (Vazyme) at a ratio of V_plasmid_:V_lipomaster 2000_ = 1:2. Subsequent detections were performed 48 hours after transfection.

### Oil Red O staining

The LMH cells transfected with the *EHHADH* gene overexpression or interference constructs were stained with Oil Red O according to the manufacturer’s instructions. The cells were fixed with 4% paraformaldehyde for 30 min and stained with Oil Red O for 30 min. After staining, the Oil Red O dye was removed and the cells were washed with 60% isopropanol. After staining with hematoxylin for 10 minutes, the cells were thoroughly washed with PBS. The stained cells were observed and imaged using an inverted phase-contrast microscope (Leica).

### Western blot

Total proteins were extracted from LMH cells transfected with the *EHHADH* gene, boiled for denaturation, quantified, and then loaded onto the gel. The proteins were separated by SDS-PAGE electrophoresis and subsequently transferred to a PVDF membrane. The membrane was blocked with TBST containing 5% non-fat milk at room temperature for 2 hours. Afterwards, it was incubated with the primary antibody Anti-Myc Tag mouse monoclonal antibody (Sangon Biotech [Shanghai]) at a dilution ratio of 1:6,000 overnight at 4°C. The membrane was washed 3 times with TBST, 5 minutes each time. Then, it was incubated with the Goat anti-mouse IgG-labeled secondary antibody (Sangon Biotech [Shanghai]) at a dilution ratio of 1:10,000 for 2 hours at room temperature, with TBST washing performed between each step. Finally, ECL substrate was added for development, and the expression level of the target protein was analyzed through band detection.

### Cell triglyceride, total cholesterol, low density lipoprotein-C, and high density lipoprotein-C assays

LMH cells were plated at 2.5×10^5^ cells/mL in six-well plates and cultured at 5% CO_2_ and 37°C for 48 hours. Cells were collected using 0.25% trypsin and lysed using ultrasonic disruption. The protein concentrations of all samples were determined using a BCA protein assay kit (Beyotime). Firstly, an appropriate amount of 25 mg/mL BSA standard solution was diluted to a final concentration of 0.5 mg/mL. Secondly, according to the number of assays, an appropriate amount of BCA working solution was prepared by mixing BCA reagents A and B at a ratio of 50:1. Thirdly, 0, 1, 2, 4, 8, 12, 16, and 20 μL of the 0.5 mg/mL BSA standard solution was added to designated standard wells of the 96-well plate, and the standard dilution buffer was added to reach a final volume of 20 μL per well. The final concentrations of the standard wells were equivalent to 0, 0.025, 0.05, 0.1, 0.2, 0.3, 0.4, and 0.5 mg/mL, respectively. Next, an appropriate volume of the sample was added to the sample wells of the 96-well plate. Per well, 200 μL of BCA working solution was added, and the plate was incubated at 37°C for 20–30 mins. Absorbance was measured at 562 nm or other wavelengths between 540 and 595 nm using a microplate reader. Finally, a standard curve was constructed based on the results of the standard samples. The protein concentration of each sample was calculated based on the standard curve and the sample volume.

Triglyceride, total cholesterol, LDL-C, and high-density lipoprotein-C (HDL-C) contents in the cell lysates were measured using the respective commercial kits (Nanjing Jiancheng Bioengineering Institute). Triglyceride, total cholesterol, and LDL-C content were measured and calculated as described above. HDL-C was measured as: the first reaction solution was prepared according to the manufacturer’s instructions, incubated at 37°C for 5 min, and the absorbance value was measured at 600 nm using a microplate reader, which was recorded as A1. The second reaction solution was added, mixed, and incubated at 37°C for 5 min. The second detection was performed under the same conditions, denoted as A2. ΔA was calculated as the difference between A1 and A2. The amount of HDL-C in the serum was calculated according to the following formula:


(3) $$\text{Concentration of HDL}-\text{C}=\frac{\left(\Delta {\text{A}}_{\text{test}}-\Delta {\text{A}}_{\text{control}}\right)}{\left(\Delta {\text{A}}_{\text{standard}}-\Delta {\text{A}}_{\text{control}}\right)}\times {\text{C}}_{\text{standard}}$$

Where C_standard_ = 0.9 mmol/L. ANOVA was used for statistical analysis of the results.

## RESULTS

### Fasting affected serum triglyceride, total cholesterol, and low density lipoprotein in chickens

**Figure 1:**
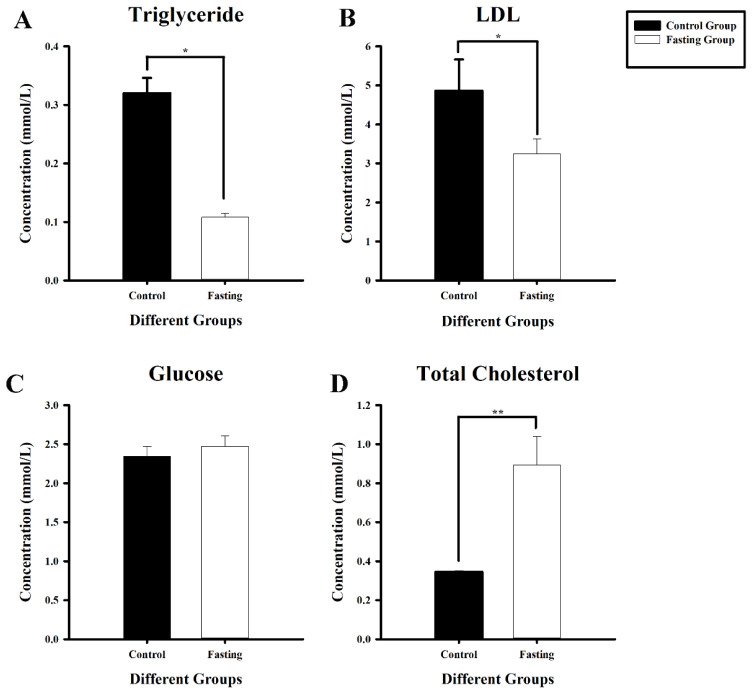
Lipid metabolism changes after fasting in the chicken liver. Blood was collected from the wing vein. Four types of serum biochemical indicators were tested after fasting: triglyceride, LDL-C, glucose, and total cholesterol after fasting. The black column represents the control group and the white column indicates the fasting group. (A) The content of triglyceride. (B) The content of LDL-C. (C) The content of glucose. (D) The content of total cholesterol. * p<0.05, ** p<0.01. LDL-C, low density lipoprotein-C.

To determine whether fasting affected liver lipid metabolism in chickens, serum triglyceride, total cholesterol, glucose, and LDL contents were measured. The results showed that the levels of TG and LDL in the fasting group were 0.33 (p = 0.004) (Figure 1A) and 0.67 (p = 0.011) (Figure 1B) times lower than those in the control group, respectively. Total cholesterol was 2.62 (p = 0.012) higher in the fasting group than that in the control group (Figure 1D). There were no significant differences in serum glucose concentrations between the two groups (Figure 1C). Therefore, it is speculated that fasting has an effect on chicken serum biochemical indexes. Moreover, fasting causes changes in lipid metabolism in chickens.

### Fasting affected the metabolome of the chicken liver

**Figure 2:**
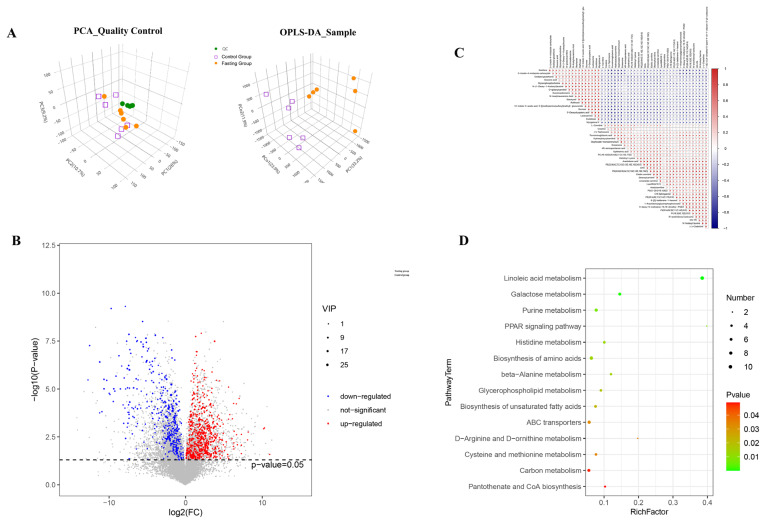
Screening of key metabolites of lipid metabolism in the liver of fasting-stressed in chickens. (A) PCA of quality control and (B) OPLSA analysis of samples. (A) PCA of quality control and OPLSA analysis of samples. (A) Green point represents quality control. The orange dots represent the fasting group; the purple box represents the control group. (B) Correlation analysis between differentially metabolized substances. Each row or column represents a metabolite, with red for positive correlation and blue for negative correlation. (C) Volcano plot used to analyze the number of up–down-regulated metabolites. Blue point indicates down-regulated metabolites; red point indicates up-regulated metabolites; gray point indicates metabolites with no difference. (D) KEGG analysis of differential metabolites. Green point indicates p<0.01; red point indicates p<0.05; larger dots represent greater numbers of metabolites involved. PCA, Principal Component Analysis; OPLS-DA, Orthogonal Partial Least Squares Discriminant Analysis; KEGG, Kyoto Encyclopedia of Genes and Genomes.

In order to further reveal the lipid substances that affect the changes of serum triglyceride and other biochemical indicators, we performed metabolomic analysis of liver of experimental chickens. QC PCA showed that, after seven-fold cross-validation, QC samples clustered closely, indicating the stability and reproducibility of this assay (Figure 2A). Simultaneously, OPLSA was performed on all samples and the results revealed differences between the control and fasting groups (Figure 2A).

There were 648 differential metabolites (VIP>1 and p-value<0.05) between the fasting and control groups. Compared to the control group, 406 differential metabolites were upregulated and 242 differential metabolites were downregulated in the fasting group (Figure 2B). These differential metabolites were classified into 12 categories, such as lipids, lipid-like molecules, organic acids and their derivatives, organoheterocyclic compounds, and organooxygen compounds. Of the 406 differential metabolites, 370 differential metabolites belonged to lipids and lipid-like molecules (Supplement 1). In the fasting group, the contents of arachidonoyl amine, isoliquiritigenin 4-O-(5’’’-O-feruloyl)-apiofuranosyl-(1’’’→2’’)-glucoside, 6-hydroxyluteolin 7-[3’’-(3-hydroxy-3-methylglutaryl)glucoside], N-docosahexaenoyl phenylalanine, calomelanol D, N-oleoyl phenylalanine, (+)-dysideapalaunic acid, 20:2(5Z, 9Z)(11Me, 15Me, 19Me), delta 8, 14 –sterol, (22S)-1alpha, 22, 25-trihydroxy-26, 27-dimethyl-23, 23, 24, 24-tetradehydrovitamin D3, and 1alpha, 25-dihydroxy-19-nor-22-oxavitamin D3 were 160 times higher than those in the control group. In contrast, the contents of sempervirenoside A, 17-beta-estradiol-3,17-beta-sulfate, sophoraflavone A, luteolin 7, 3’, 4’-triglucuronide, embinin, 20:0 campesterol ester, malvidin 3-glucoside-5-(6-acetylglucoside), and phellodensin F in the fasting group were 160 times lower than those in the control group.

Pearson’s correlation analysis was used to analyze the correlation between the different metabolites (Figure 2C). There were 14 groups of metabolites with correlation coefficients greater than 0.99 (Table 2).

KEGG analysis of the differential metabolites revealed 73 differential KEGG pathways between the fasting and control groups (Figure 2D). Examples of these differentially expressed KEGG pathways include arachidonic acid metabolism, unsaturated fatty acid biosynthesis, linoleic acid metabolism, fatty acid biosynthesis, and fatty acid degradation.

### Fasting affected the expression of lipid metabolism-related genes in the chicken liver

**Figure 3:**
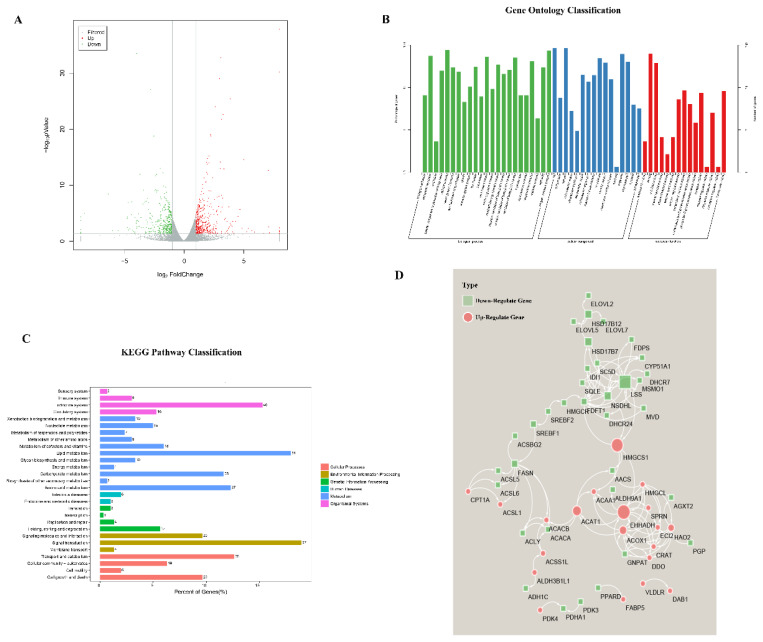
Screening of DEGs for lipid metabolism in fasting-stressed chicken livers. (A) Volcano plot, used to analyze the number of up–down-regulated genes. The screening was carried out under the condition of Log_2_ (Fold Change)>1 and p<0.05. One point represents a gene; the red point represents up-regulated genes; the green point represents down-regulated genes; the gray point was genes with no significant difference. (B) GO analysis of DEGs. Green column indicates biological process; blue column indicates the cellular component; red column indicates molecular function; Higher columns represent more genes. (C) KEGG analysis of DEGs. Blue column represents the lipid metabolism, including54 pathways. (D) PPI analysis of part of the DEGs. Green square indicates down-regulated genes; red circle indicates up-regulated genes; a larger trait represents more genes that interact with it. DEG, differentially expressed gene; GO, Gene Ontology; KEGG, Kyoto Encyclopedia of Genes and Genomes; PPI, protein–protein interaction.

**Figure 4:**
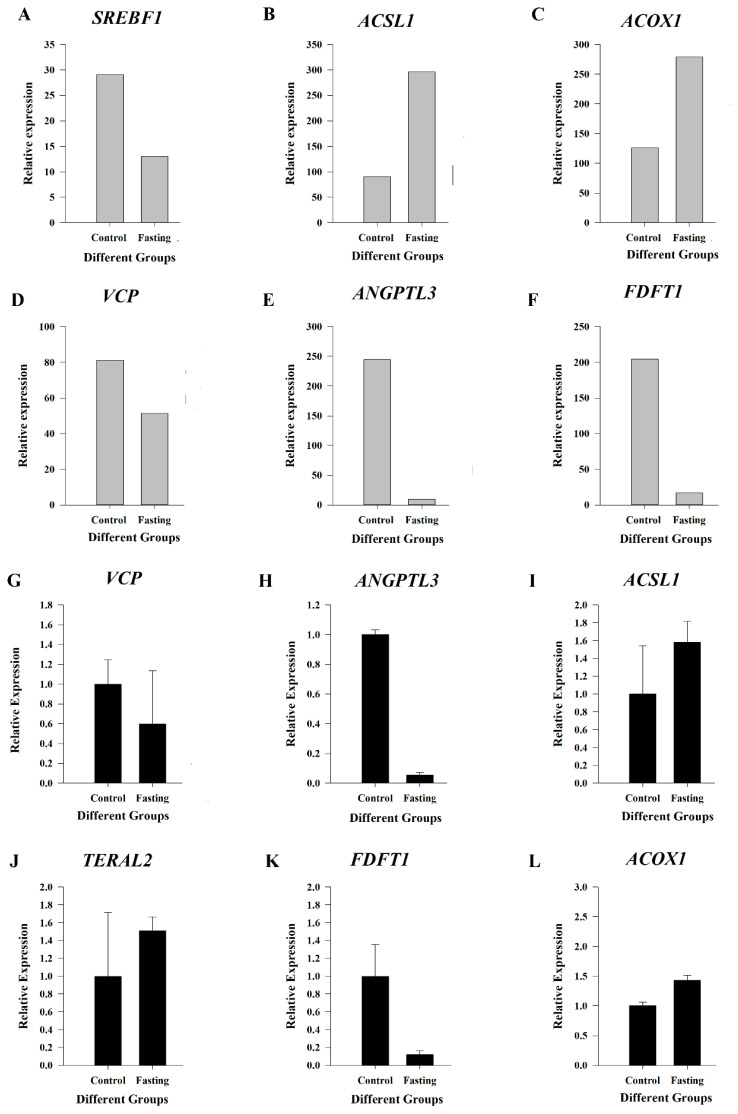
Quantitative PCR-based verification of the expression of representative genes. The expressions of six genes, *Srebf1*, *Acsl1*, *Acox1*, *VCP*, *ANGPTL3*, *FDFT1* were consistent with the sequencing results. PCR, polymerase chain reaction.

To analyze the the genes that affect the changes of metabolic substances in the liver, livers of 2-week-old chickens were isolated after fasting for 72 hours and subjected to RNA-seq. Using the criteria |log_2_ (fold change)|>1 and p<0.05, 849 DEGs were identified by comparing the fasting to the control group, including 438 upregulated and 411 downregulated DEGs in the fasting group (Figure 3A). GO analysis was performed to determine the biological processes, molecular functions, and cellular components enriched in the DEGs (Figure 3B). The analysis revealed that 78 DEGs were involved in lipid metabolism or fatty acid metabolism (Table 3). PPI analysis of these 78 DEGs revealed more than 90 interactions between 20 DEGs, including *FASN*, *SCD*, *ACSL5*, *ACACA*, *ACACB*, *SREBF1*, *SREBF2*, *AACS*, *HMGCS1*, *ANGPTL3*, *FDFT1*, *ACOX1*, *EHHADH*, *CPT1A*, hydroxyacid oxidase (*HAO2*), *ACAA1*, *ALDH3B1L*, *ACSL1*, *ECI2*, and lanosterol synthase (*LSS*) (Figure 3D). Among them, the top three interacting genes were *HMGCS1*, *EHHADH* and *LSS*. KEGG analysis of the DEGs revealed the enrichment of 135 pathways, including fatty acid degradation, fatty acid biosynthesis, arachidonic acid metabolism, and linoleic acid metabolism (Figure 3C). RNA-seq data were verified using RT-qPCR (Figure 4).

### Associating differential metabolites with differentially expressed genes

**Figure 5:**
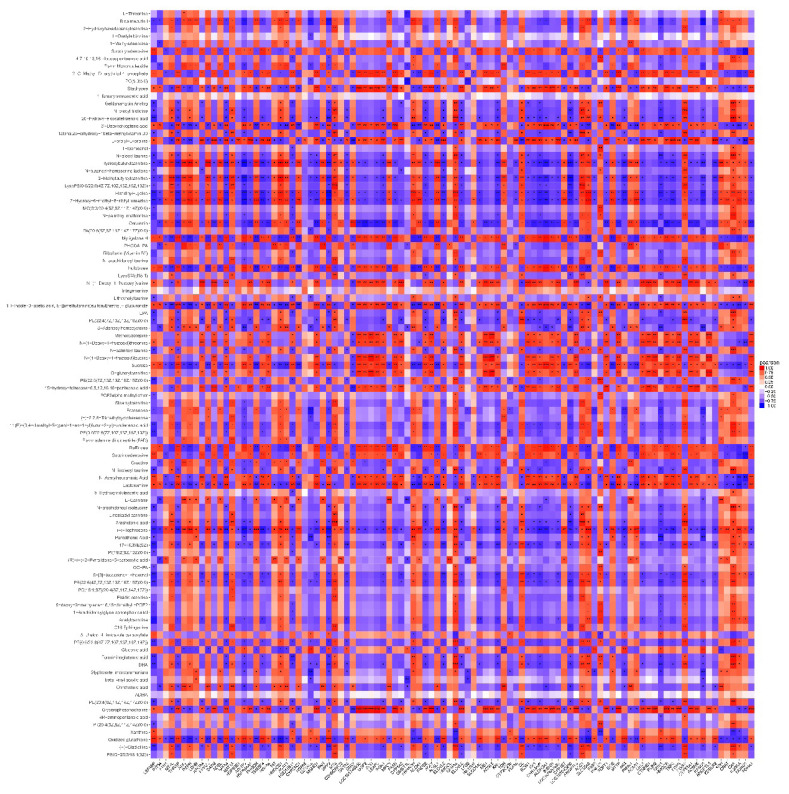
Correlation analysis between differential genes and metabolites. Each row represents a differential metabolite and each column represents a differential gene. Darker red represents a correlation coefficient closer to 1; darker purple represents a correlation coefficient closer to −1. * p<0.05, ** p<0.01, *** p<0.001. The absence of * between differential metabolites and DEGs indicates that they are not correlated. DEG, differentially expressed gene.

**Figure 6:**
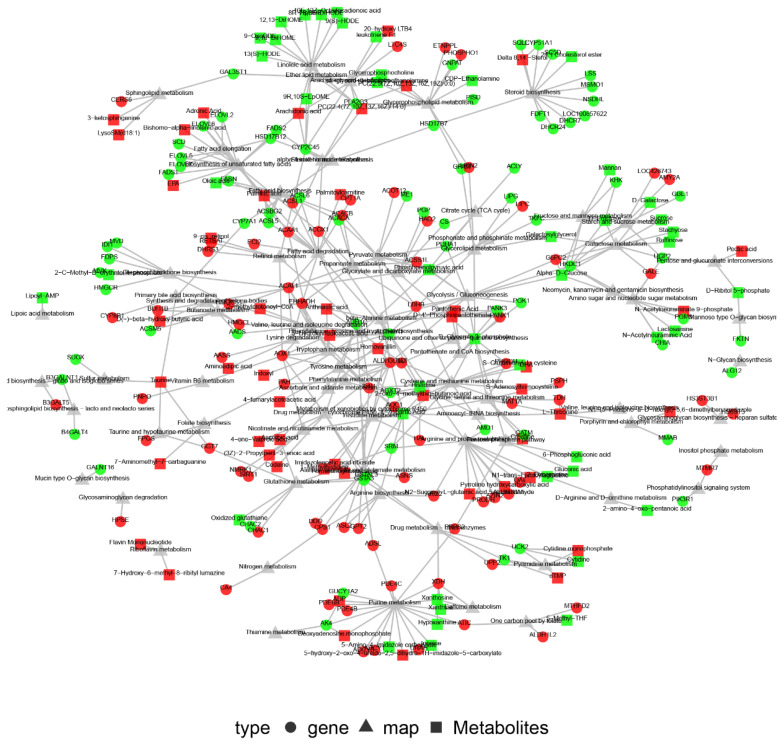
Network interaction analysis of differential genes, differential metabolites, and pathways. (A) KGML analysis of all DEGs, all differential metabolites, and pathways. Circles represent genes, squares represent metabolites, and triangles represent pathways. Red indicates up-regulated expression, green indicates down-regulated expression, and gray represents only material but not expression. KGML, KEGG Markup Language; DEG, differentially expressed gene.

In order to further explore the relationship between lipid substances and candidate genes in the liver, the combined analysis of metabolomics and transcriptomics was performed. Pearson’s correlation analysis was used to identify DEGs associated with differential metabolites in the chicken liver. We found that 101 DEGs were correlated with 99 differential metabolites (p<0.05) (Figure 5). Of these correlations, the *EHHADH* gene was associated with 54 differential metabolites, whereas the 3-hydroxy-3-methylglutaryl-CoA synthetases (*HMGCS1*) gene was associated with 53 differential metabolites. KGML analysis (Figure 6) revealed that the *EHHADH* gene was associated with various metabolic pathways, such as fatty acid degradation and beta-alanine and propanoate metabolism. Venn analysis revealed 63 common pathways between the transcriptome and metabolome, which were primarily involved in lipid metabolism, steroid biosynthesis, and arachidonic acid metabolism.

Combining these analyses, we found the *EHHADH* gene was a hub gene regulated by fasting in the chicken liver. PPI analysis showed that *EHHADH* interacted with 94 other genes and was associated with 54 metabolites.

### The *EHHADH* gene reduced lipid deposition in the LMH cell line

**Figure 7:**
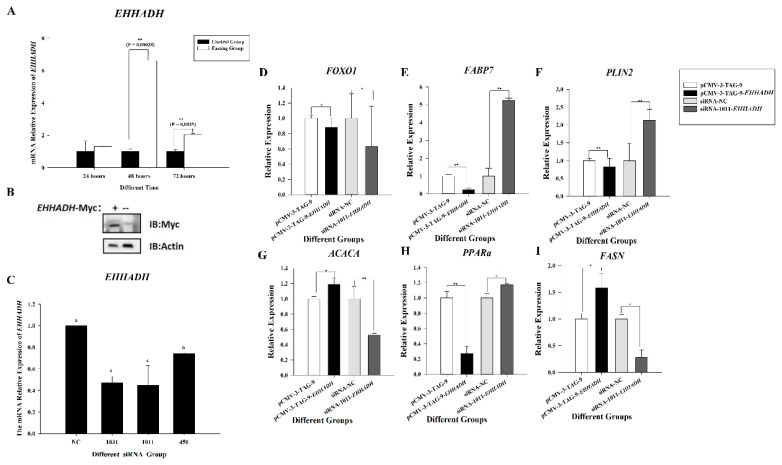
Lipid metabolism genes changed after interfering with or overexpressing the *EHHADH*. (A) The mRNA expression of *EHHADH* after fasting for 24, 48, and 72 h in chicken liver. Black column indicates control and white indicates fasting group content. ** indicates p<0.05. (B) Identification of recombinant plasmids. The identification was carried out using the MYC tag in the pCMV-3-TAG-9 vector. The left lane marked with "+" represents the pCMV-3-TAG-9-*EHHADH* recombinant plasmid, while the right lane represents LMH cells. “IB” means immunoblotting. The upper band was MYC, and the under band was actin. (C) Three-group siRNA interference efficiency detection. The x-axis indicates the three groups of siRNA and the control: 1011: siRNA-1011, 1831: siRNA-1831, 450: siRNA-450, and NC: non-specific control. The y-axis indicates the mRNA expression content of *EHHADH*. Lowercase letters, a, b, and c indicates p<0.05. The same letters represent insignificant differences and different letters represent significant differences. (D–I) The changes in lipid metabolism-related genes after interfering with or overexpressing the *EHHADH* gene. In the overexpression treatment, the control group is the empty vector-transfected group; in the interference group, the control group is the siRNA-NC group. “*” means p<0.05, “**” was p<0.01.

To validate the role of the *EHHADH* gene in lipid deposition in chicken liver, we determined the effect of fasting for 24, 48, and 72 h on the mRNA expression of *EHHADH* in the chicken liver. The expression of the *EHHADH* gene in the fasting group was 6.12 times (p = 0.00028) and 2.13 times (p = 0.0035) higher than that in the control group after 48 and 72 h of fasting, respectively (Figure 7A).

To further explore the function of this gene, we constructed the pCMV-3-TAG-9-*EHHADH* overexpression plasmid and the fragment that interferes with the *EHHADH* gene, respectively. Compared with the control group (pCMV-3-TAG-9), the recombinant plasmid showed a MYC band, while the control group did not, indicating that the overexpression recombinant plasmid was successfully constructed (Figure 7B). We designed three siRNAs and all three siRNAs inhibited the expression of *EHHADH* gene, of which siRNA-1011-*EHHADH* had the highest knockdown efficiency (Figure 7C). Therefore, siRNA-1011-*EHHADH* was used in subsequent experiments.

After siRNA-1011-*EHHADH* and pCMV-3-TAG-9-*EHHADH* were transfected into LMH cells for 48 h respectively, *Fabp7*, *ACACA*, *FASN*, *PPARa* and *PLIN2* gene were showed significant changed (Figures 7D–7I). When the *EHHADH* gene was overexpressed, the mRNA expressionlevels of *ACACA* and *FASN* were significant up-regulated. Compared with the control group, in the overexpression group, the expression level of *ACACA* was upregulated by 1.19-fold (p = 0.035), and the expression level of *FASN* was upregulated by 1.58-fold (p = 0.025). After the *EHHADH* gene was interfered, the expression levels of these two genes were significant down-regulated. After interference, compared with the control group, the expression levels of both genes in the overexpression group decreased by 47%. *Fabp7*, *Plin2* and *PPARα* showed opposite trends. After overexpression of the *EHHADH* gene, the expression levels of these three genes were significantly down-regulated (in the overexpression group, the expression level of *FABP7* was 0.24 times that of the control group, the expression level of *Plin2* was 0.82 times that of the control group, and the expression level of *PPARα* was 0.27 times that of the control group). After interference with the *EHHADH* gene (in the interference group, the expression level of *FABP7* was 5.23 times that of the control group, the expression level of *Plin2* was 2.13 times that of the control group, and the expression level of *PPARα* was 1.17 times that of the control group), the expression levels of these three genes were significantly increased.

**Figure 8:**
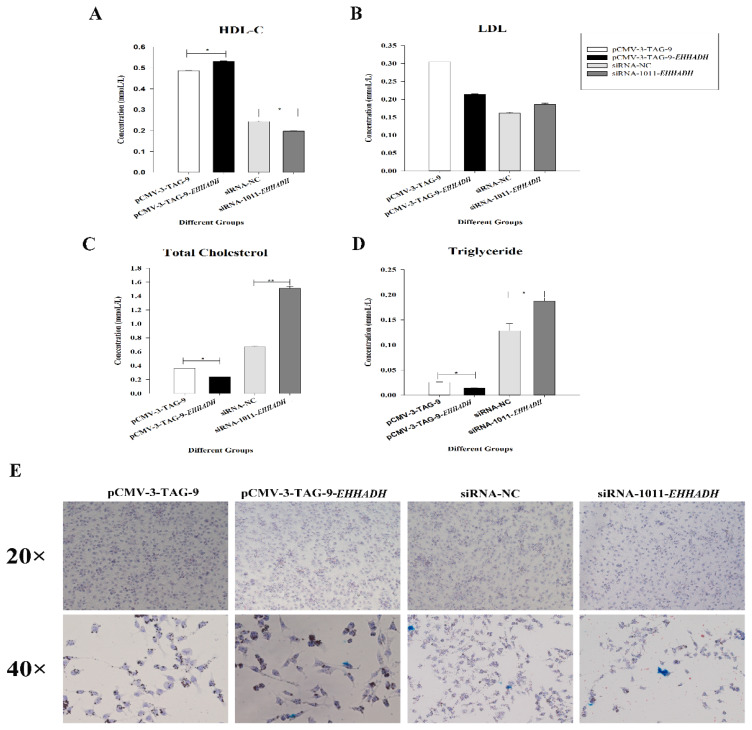
Lipid deposition in LMH cells increased after interfering with or overexpressing *EHHADH*. (A–D) Detection of intracellular biochemical parameters in LMH cells after interfering with or overexpressing *EHHADH*. The contents of the four biochemical indicators in LMH cells were detected after overexpression or interference of the *EHHADH* gene, include HDL-C (A), LDL (B), total cholesterol (C) and triglycerides (D). The white bars represent the overexpression control group (transfected with pCMV-3-TAG-9), the black bars represent the *EHHADH* overexpression group, the light gray bars represent the interference control group, and the dark gray bars represent the *EHHADH* interference group. * p<0.05, ** p<0.01. (E) Oil Red O staining after overexpression or interference of the *EHHADH* gene. We recorded two bright field visions: 200× and 400×. pCMV-3-TAG-9: overexpression control group, LMH cells transfected with pCMV-3-TAG-9 (empty plasmid), pCMV-3-TAG-9-*EHHADH*: overexpression group, LMH cells transfected with pCMV-3-TAG-9-*EHHADH*. SiRNA-NC: interference control group, LMH cells transfected with non-specific control, siRNA-1011-EHHADH: interference group, LMH cells transfected with siRNA-1011. Bar value under 20× is 100 μm, and 40× is 60 μm. HDL-C, high density lipoprotein-C; LDL, low density lipoprotein.

Oil red O staining results showed that lipid deposition decreased after overexpression of the *EHHADH* gene, while it increased after interference with the *EHHADH* gene (Figure 8E).

Additionally, after overexpression of the *EHHADH* gene, the content of HDL-C in LMH cells increased, while it decreased after interference. On the contrary, overexpression of the *EHHADH* gene led to a reduction in the contents of triglycerides and total cholesterol in LMH cells, and both contents increased after interference. Further demonstrating the role of the *EHHADH* gene in lipid deposition in hepatocytes (Figures 8A–8D).

## DISSCUSSION

In this study, we identified 78 lipid metabolism-related genes (Table 3) and 370 metabolites (Supplement 1) through RNA-seq and metabonomic analyses of chicken livers subjected to a 72-hour fast. We examined gene and metabolite profiles during fasting and conducted a combined analysis, revealing that *EHHADH* is the hub gene involved in lipid metabolism in the chicken liver. Oil Red O staining and RNAi techniques were used to elucidate the regulatory mechanism of *EHHADH* in lipid metabolism in chicken LMH cells, offering insights relevant to the poultry industry.

We found the levels of TG in the fasting group were 0.33 (p = 0.004) (Figure 1A) times lower than those in the control group. This result is consistent with Wu et al, which found that triglyceride content decreased significantly with the increase of fasting time [8]. This indicates that the lipid metabolism in the liver of chickens changes during starvation. During starvation, the metabolic pattern of the chicken’s liver changes. The liver will increase the beta-oxidation of fatty acids to generate more energy. At the same time, the activity of key enzymes involved in triglyceride synthesis in the liver is inhibited, while the activity of enzymes involved in gluconeogenesis is enhanced. This is because chickens need to maintain the stability of blood glucose levels through gluconeogenesis to meet the needs of tissues such as the brain that rely on glucose for energy supply. Therefore, the raw materials originally used for triglyceride synthesis, such as dihydroxyacetone phosphate, will flow more towards the gluconeogenesis pathway, resulting in a decrease in the amount of triglycerides synthesized by the liver, and consequently, the triglycerides secreted into the serum also decrease accordingly [8].

Metabonomics, as a downstream approach that closely mirrors biological phenotypes, can be used to explore the patterns of change in stimulated and small molecules. The liver plays a key role in lipid metabolism in poultry. Therefore, we conducted metabolomic analysis comparing fasting and non-fasting metabolites in chicken liver. An increase in the contents of fatty amides, polyketides, prenol lipids, sterol lipids, and glycerophospholipids was apparent, whereas a decrease was noticed in the flavonoids and sterols after fasting. Fatty acid amides are lipid bioregulators formed by the amidation of long-chain saturated and unsaturated fatty acids with their corresponding amines [9]. Specific fatty acid amides interact with cannabinoid and vanilloid receptors, often referred to as “endocannabinoids” or “endovanilloids,” leading to pain reduction and regulating lipid and glucose metabolism, vasodilation, cardiac function, and inflammation [10].

To further reveal the mechanisms underlying the metabolic changes, transcriptome sequencing was performed. Sixty chickens were divided into control and fasting groups and observed for 72 hours followed by transcriptome sequencing. This analysis revealed various DEGs (Table 3), including *HMGCS1*, *LSS*, *EHHADH*, and *HAO2* as the hub genes, which we intended to validate further. HMGCS include mitochondrial (HMGCS2) and cytoplasmic (HMGCS1) forms [11]. Studies indicate 65% amino acid similarity between hamster cytoplasmic and mouse mitochondrial HMGCS, though they show distinct regulatory mechanisms. For example, cholesterol administration decreases cytoplasmic HMGCS while increasing mitochondrial HMGCS. Mitochondrial HMGCS mediates ketone body biosynthesis, whereas cytoplasmic HMGCS participates in cholesterol biosynthesis [12,13]. LSS is a key enzyme in the cholesterol synthesis pathway that catalyzes the conversion of (S) -2, 3-eposqualene into lansterol, the first sterol produced in cholesterol synthesis. Inhibiting LSS activity affects downstream sterol production but spares upstream products, such as farnesyl pyrophosphate and geranyl pyrophosphate. These characteristics suggest that LSS may have fewer side effects than statins, although the specific molecular mechanism remains unclear [14,15]. HAOs are a class of peroxisomal oxidases with three isoforms. *HAO2* is primarily expressed in the human liver and kidney and catalyzes the oxidation of hydroxylate-containing fatty acids to keto acids and hydrogen peroxide [16].

Combined analysis of DEGs, differential metabolites, and candidate pathways revealed that *HMGCS1* and *EHHADH* participates in various signaling pathways. However, in subsequent studies, it was found that the *HMGCS1* gene is only expressed in liver cancer tissues, and the mRNA expression level of this gene in normal chicken livers is extremely low. Therefore, we chose the *EHHADH* gene for research. *EHHADH* invloved in various signaling pathways, including beta-alanine and butanoate metabolism; fatty acid and lysine degradation; propionate metabolism and tryptophan metabolism; and valine, leucine, and isoleucine degradation; and exerts a regulatory effect on the metabolite 2-methycrotonoy-CoA. Therefore, we further investigated the *EHHADH* gene, a member of the 3-hydroxyacyl-CoA dehydrogenase family [17]. *EHHADH* catalyzes two steps in fatty acid oxidation [18,19], and its deficiency disrupts normal fatty acid metabolism [20]. Zhao et al found that the *EHHADH* protein possesses four acetylated lysine residues (Lys165, Lys171, Lys346, and Lys584) and confirmed that it is acetylated. This observation indicates that most of *EHHADH* is acetylated, and its acetylation can be dynamically regulated *in vivo* [21]. In the current study, after silencing the *EHHADH* gene using siRNA or over espressed *EHHADH*, the mRNA expression of *ACACA*, *FABP7*, *FASN*, and *PPARa* were significant changed. Further, the contents of triglycerides, total cholesterol and HDL-C and lipid deposition increased, after interfering with the *EHHADH* gene indicating that the *EHHADH* gene had an inhibitory effect on lipid deposition. This result is consistent with those reported by Yang et al [22] and Chen et al [23]. Yang et al found that knockout of *EHHADH* substantially inhibited the SREBF and PPAR signaling pathways in the zebrafish liver, resulting in increased liver lipid deposition [22]. Chen et al. showed that liver deposition increased after arsenic exposure in mice, and the triglyceride content or mRNA expression of *EHHADH* was upregulated [23]. Our findings, along with these results, indicate that *EHHADH* plays an important role in lipid regulation in the liver.

## CONCLUSION

In conclusion, transcriptome data and metabolomics analysis identified several hub genes and metabolites involved in the lipid metabolism of the chicken liver. Fasting induced substantial changes in lipid metabolism. Importantly, *EHHADH* was identified as a key factor in reducing lipid deposition in the chicken liver.

## Figures and Tables

**Table 1:** The list of primer for RT-qPCR

Gene	Primer
*VCP*	Forward: GGCAGCTTCAGGTTCCCAT Reverse: AAGTAGCCGAGAGTCCACTG
*ANGPTL3*	Forward: ATGCACAAACTGGAAGGTGC Reverse: CGAAAGCTTCTGAGCCGTTG
*ACSL1*	Forward: GCTGCCGGAGGTTCCAG Reverse: CACCAAAACCCACCAGGGTA
*FDFT1*	Forward: GAGGACTTCCCAACGATCTCC Reverse: ACCGATCCCACTCACTCTGA
*ACOX1*	Forward: GGCGAAAGGAGATCGAGGC Reverse: GCCGTCCACGATGAACAAAG
*SREBF1*	Forward: GACCATCCTGGCCACAGTAC Reverse: TCTCAATGGCATTGTGGGCT
*EHHADH*	Forward: CAACGCGCTGAGTTTGACAG Reverse: CCCAAGCCAAGACCCTGTTT
*PLIN2*	Forward: ATTTCTTTGCGGGCTCTGTA Reverse: ACTCCTTTCTCTGCTATCTCACA
*FOXO1*	Forward: GCTATGAACCCGTATGCCCC Reverse: CCATGTCACAGTCCAACCGT
*FASN*	Forward: GGGTATGGTGAACTGCCTCC Reverse: CTGCAATGGGAGATGCCTGA
*FABP7*	Forward: CGACGACAGGAACTGCAAATC Reverse: CACCACATCACCAAAGGTAAGAGT
*PPARa*	Forward: GGGATGCTGGTAGCCTATGG Reverse: AGACCAGGACGATCTCCACA
*ACACA*	Forward: CCACCCAAACAGAATGTCCT Reverse: GGGGAGGAGAGTCGCAAAAA
*β-actin*	Forward: CACGGTATTGTCACCAACTG Reverse: ACAGCCTGGATGGCTACATA
*EHHADH*-CDS	Forward (BamH I): CGGATCCATGGCGCAGTACGCGAGGGGCG Reverse (Hind III): CAAGCTTTCACAGCTTGTTGCTCTGCCTG

RT-qPCR, reverse transcription quantitative polymerase chain reaction.

**Table 2:** The list of correlation analysis of differential metabolites (R^2^>0.99)

Metabolite 2	Metabolite 1									

	Xanthine	N-(1-Deoxy-1-fructosyl)leucine	Raffinose	1H-Indole-3-acetic acid, 5-[{(methylamino)sulfonyl}methyl]-glucuronide	Sucrose	3'-Deoxydryopteric acid	Inulobiose	PE(0:0/22:6(4Z,7Z,10Z,13Z,16Z,19Z))	9-Deoxy-9-methylene-16,16-dimethyl -PGE2	PI (20:4 (5Z,8Z,11Z,14Z) / 0:0)
5-Ureido-4-imidazole carboxylate	0.9944									
O-glutarylcarnitine		0.9974								
Sucrose			0.9905	0.9975						
3'-Deoxydryopteric acid				0.9957	0.99836					
Raffinose					0.9905					
1H-Indole-3-acetic acid,5-[{(methylamino)sulfonyl}methyl]-lucuronide					0.9975	0.9957				
Myrigalone H							0.9952			
DHA								0.9948		
1-Arachidonoylglycerophosphoinositol									0.9946	0.9942
PI(18:2(9Z,12Z)/0:0)										0.9935

**Table 3:** Screening list of candidate DEGs in fasting group vs. control group

Gene	Control group expression	Fasting group expression	Fold change	log_2_ (fold change)	p-value	Regulation
*ACOT12*	0.026987	0.708862	29.04409	4.860173	0.004736	Up
*ACACB*	0.047886	1.186098	25.88681	4.694145	2.30E-06	Up
*CPT1A*	5.760847	63.73493	10.7081	3.42063	0.000143	Up
*LOC100858796*	2.487637	11.36478	4.863241	2.281918	1.16E-09	Up
*PDK4*	8.83856	44.61713	4.821741	2.269554	1.68E-05	Up
*NR1D1*	0.646435	3.132487	3.935611	1.976588	0.001048	Up
*ACSL1*	90.65093	295.9953	3.23066	1.691829	5.14E-12	Up
*ACAA1*	51.07163	154.883	2.962422	1.566777	7.21E-11	Up
*CPS1*	0.366583	1.062272	2.881306	1.526723	3.58E-06	Up
*FABP3*	0.978343	2.34158	2.862298	1.517174	0.012755	Up
*ALDH3B1L1*	0.174648	0.620813	2.850197	1.511062	0.012272	Up
*BDH1B*	1.18026	3.065077	2.508423	1.326781	0.003261	Up
*CERS6*	0.462496	1.203402	2.408279	1.268003	0.004318	Up
*NR1D2*	8.20019	20.97353	2.403723	1.265271	5.58E-07	Up
*FTO*	0.436303	0.973968	2.344518	1.229291	0.006516	Up
*PID1*	1.204667	2.806067	2.313865	1.210305	0.000533	Up
*VLDLR*	1.061639	2.47418	2.313807	1.210268	0.000144	Up
*ECI2*	65.20037	154.6377	2.311855	1.209051	2.70E-07	Up
*IRS2*	1.838263	4.319027	2.282537	1.190638	0.00583	Up
*CREBL2*	11.51866	25.93963	2.266119	1.180224	4.27E-06	Up
*ACOX1*	126.2333	278.9957	2.263281	1.178416	7.55E-07	Up
*LOC417013*	29.314	62.0175	2.230204	1.157175	0.002558	Up
*GNLY*	201.0313	412.686	2.212072	1.145398	0.000118	Up
*HAO2*	21.84147	48.92617	2.165813	1.114909	8.11E-05	Up
*EHHADH*	137.6077	297.182	2.139445	1.097237	2.42E-06	Up
*ADGRF5*	0.8085	1.71358	2.115675	1.081118	0.000299	Up
*FABP5*	2.73297	5.793907	2.112496	1.078949	0.001302	Up
*EXFABP*	6.127133	13.05587	2.10238	1.072023	0.009832	Up
*CRAT*	47.12513	100.9184	2.076834	1.054386	8.94E-06	Up
*PLCL1*	0.107044	0.243383	2.049949	1.035588	0.041875	Up
*LTC4S*	0.725282	1.573113	2.039922	1.028514	0.00582	Up
*ORMDL1*	18.3324	8.87902	0.49939	−1.00176	0.001111	Down
*PLEKHA8*	2.29296	1.191747	0.488547	−1.03343	0.00461	Down
*PDK3*	30.34903	15.72317	0.486749	−1.03875	0.016004	Down
*ELOVL7*	2.110833	1.067735	0.4803	−1.05799	0.002348	Down
*GNPAT*	16.96727	8.46907	0.477066	−1.06774	2.81E-05	Down
*MCOLN3*	0.281593	0.133183	0.475881	−1.07133	0.0027	Down
*HSD17B12*	87.24003	41.4001	0.469953	−1.08941	0.000126	Down
*ELOVL2*	74.71407	36.5768	0.466689	−1.09947	0.001264	Down
*PISD*	10.53344	4.933723	0.465234	−1.10397	0.009094	Down
*SREBF1*	29.1286	13.00843	0.46452	−1.10619	5.76E-06	Down
*LPAR3*	1.887487	0.928238	0.462016	−1.11399	0.008684	Down
*MTTP*	145.645	69.72107	0.458224	−1.12587	0.004278	Down
*LBFABP*	8,334.623	3,798.57	0.434616	−1.20219	2.82E-07	Down
*PRELID3A*	2.405293	1.073383	0.405936	−1.30067	0.000499	Down
*FADS1*	68.89273	29.09797	0.40157	−1.31628	0.008751	Down
*MELTF*	16.39407	6.60493	0.395045	−1.33991	0.004759	Down
*ACSM5*	112.7307	44.69063	0.378387	−1.40206	0.005455	Down
*LOC112530174*	3.910153	1.949377	0.376452	−1.40946	4.46E-05	Down
*INSIG1*	199.568	79.23733	0.375857	−1.41174	0.003766	Down
*LOC107049564*	0.142441	0.054634	0.370963	−1.43065	0.039228	Down
*SEC14L3*	13.88707	4.648357	0.369313	−1.43709	0.040214	Down
*EPN3*	49.72943	17.53067	0.362579	−1.46363	8.70E-05	Down
*ACSL5*	242.9483	91.61523	0.361943	−1.46616	0.045425	Down
*SREBF2*	67.8193	22.18007	0.332855	−1.58703	9.53E-07	Down
*LIPG*	1.253781	0.341898	0.278215	−1.84573	9.28E-05	Down
*PGP*	28.72657	8.01743	0.274782	−1.86364	0.008036	Down
*PLPPR5*	0.212219	0.045526	0.270418	−1.88674	0.001727	Down
*SERINC2*	7.692383	1.935221	0.266016	−1.91042	2.59E-08	Down
*ELOVL6*	294.9077	81.54667	0.261956	−1.9326	0.000231	Down
*ANXA13*	1.435063	0.320753	0.260604	−1.94007	2.15E-05	Down
*LDLR*	8.238497	1.8177	0.254991	−1.97148	3.80E-07	Down
*ELOVL5*	305.799	78.27647	0.247209	−2.0162	1.78E-07	Down
*PLIN2*	153.6528	40.42073	0.245511	−2.02614	0.006011	Down
*FADS2*	324.9277	78.84733	0.233865	−2.09625	0.000274	Down
*LSS*	75.95853	16.01813	0.208733	−2.26027	0.000314	Down
*ACACA*	105.7296	18.287	0.174557	−2.51823	1.74E-19	Down
*GRHL1*	0.202372	0.03687	0.146775	−2.76832	0.000409	Down
*ACSL6*	0.245303	0.038523	0.128679	−2.95815	2.11E-06	Down
*NSDHL*	99.10147	13.23584	0.128453	−2.96069	1.24E-07	Down
*SPTSSB*	0.361004	0.035085	0.094796	−3.39903	0.003369	Down
*FDFT1*	204.668	17.0023	0.082175	−3.60515	6.01E-05	Down
*FASN*	453.4297	33.4005	0.072124	−3.79338	1.20E-05	Down
*PLA2G3*	14.58487	0.920773	0.06455	−3.95345	0.000197	Down
*ACSBG2*	108.5219	5.144823	0.046554	−4.42496	1.86E-05	Down
*AACS*	62.84057	2.52997	0.038174	−4.71127	0.003361	Down
*ANGPTL3*	244.323	9.529957	0.037117	−4.75179	7.70E-05	Down
*SCD*	539.958	1.600277	0.003138	−8.31583	1.02E-05	Down

DEG, differentially expressed gene.

## SUPPLEMENTARY MATERIAL

**Supplement 1.** The result of differential metabolites.
