# Supplementary material for: Transcriptome and metabolite profiling reveals the mechanism of hepatic lipid metabolism during fasting in chicken
Source: Anim Biosci. 2025 Aug 12;39(1):250014. doi: 10.5713/ab.25.0014 (PMC12754495; doi:10.5713/ab.25.0014)
Supplement: Supplementary file 1 [file ab-25-0014-Supplementary-1.pdf]

Supplement 1. The result of differential metabolites

ID	Metabolites	Class	VIP	P-value	log_2(FoldChange)	Fold Change
13.18_803.6036n	PS(O-20:0/18:1(9Z))	Glycerophospholipids	20.37	0.02	0.52	1.43
13.03_304.2406n	(-)-Cladielline	Prenol lipids	15.98	0.00	1.80	3.48
10.93_620.2955n	PI(20:4(5Z,8Z,11Z,14Z)/0:0)	Glycerophospholipids	15.66	0.01	2.74	6.70
0.76_257.1030n	Glycerophosphocholine	Glycerophospholipids	14.90	0.00	-2.40	0.19
12.84_328.2403n	DHA	Fatty Acyls	11.48	0.00	2.44	5.42
10.74_525.2854n	PE(0:0/22:6(4Z,7Z,10Z,13Z,16Z,19Z))	Glycerophospholipids	10.98	0.00	2.67	6.38
10.99_426.3576m/z	Elaidic carnitine	Fatty Acyls	10.68	0.01	3.14	8.80
13.03_779.5440n	PC(16:1(9Z)/20:4(8Z,11Z,14Z,17Z))	Glycerophospholipids	9.69	0.00	1.96	3.90
10.76_501.2842n	PE(20:4(8Z,11Z,14Z,17Z)/0:0)	Glycerophospholipids	9.43	0.03	1.18	2.26
10.69_424.3417m/z	Linoelaidyl carnitine	Fatty Acyls	8.96	0.01	3.75	13.49
10.65_525.2842n	PE(22:6(4Z,7Z,10Z,13Z,16Z,19Z)/0:0)	Glycerophospholipids	8.94	0.01	2.14	4.41
10.72_620.2955n	1-Arachidonoylglycerophosphoinositol	Glycerophospholipids	8.69	0.01	1.80	3.48
0.80_118.0855m/z	4R-aminopentanoic acid	Fatty Acyls	8.51	0.01	0.57	1.48
2.29_375.1146m/z	(+)-Tephrosone	Polyketides	8.45	0.00	3.84	14.33

10.72_361.2735m/z	9-deoxy-9-methylene-16,16-dimethyl -PGE2	Fatty Acyls	8.28	0.01	1.68	3.20
0.85_341.1323n	Lactosamine	Fatty Acyls	8.26	0.00	-2.34	0.20
13.03_417.3235n	N-arachidonoyl isoleucine	Fatty Acyls	7.58	0.00	4.90	29.90
10.88_596.2953n	PI(18:2(9Z,12Z)/0:0)	Glycerophospholipids	7.22	0.01	2.23	4.70
10.61_595.2908m/z	OG-PA	Glycerophospholipids	7.11	0.03	1.87	3.66
12.85_256.2636m/z	C16 Sphinganine	Sphingolipids	6.99	0.02	1.15	2.22
13.03_303.2344m/z	Arachidonic acid	Fatty Acyls	6.79	0.01	2.01	4.02
11.79_387.2436n	N-linoleoyl taurine	Fatty Acyls	6.77	0.00	2.45	5.48
0.87_286.1274n	Myrigalone H	Polyketides	6.45	0.00	-4.93	0.03
11.33_428.3726m/z	Stearoylcarnitine	Fatty Acyls	6.37	0.00	3.31	9.89
13.19_348.2667n	11(E)-(3,4-dimethyl-5-(pent-1-en-1-yl)furan-2-yl)-undecanoic acid	Fatty Acyls	6.17	0.00	2.62	6.15
2.27_275.1344n	O-glutarylcarnitine	Fatty Acyls	5.87	0.00	-1.84	0.28
10.69_448.3414m/z	LysoSM(d18:1)	Sphingolipids	5.75	0.00	3.94	15.35
2.04_248.1490m/z	Hydroxybutyrylcarnitine	Fatty Acyls	5.74	0.00	3.92	15.11

0.84_377.0852m/z	3'-Deoxydryopteric acid	Polyketides	5.46	0.00	-5.02	0.03
13.18_262.2294n	6-[3]-ladderane-1-hexanol	Fatty Acyls	5.45	0.02	0.84	1.78
1.20_335.0493m/z	Erosenone	Polyketides	5.19	0.00	1.30	2.46
0.89_609.1881m/z	Isoliquiritigenin 2'-glucosyl-(1->4)-rhamnoside	Polyketides	4.89	0.00	-7.18	0.01
11.83_410.2377m/z	N-arachidonoyl taurine	Fatty Acyls	4.75	0.01	4.01	16.07
11.12_527.2999n	PE(22:5(7Z,10Z,13Z,16Z,19Z)/0:0)	Glycerophospholipids	4.73	0.01	1.76	3.40
9.76_483.3011n	Lithocholyltaurine	Sterol Lipids	4.73	0.00	2.05	4.13
10.28_437.3480m/z	N-oleoyl histidine	Fatty Acyls	4.66	0.00	3.54	11.62
13.18_298.2507n	17-HOME(9Z)	Fatty Acyls	4.62	0.02	0.70	1.62
10.44_413.3480m/z	1alpha,25-dihydroxy-1beta-methylvitamin D3	Sterol Lipids	4.60	0.00	3.23	9.40
11.03_528.3077m/z	PE(0:0/22:5(7Z,10Z,13Z,16Z,19Z))	Glycerophospholipids	4.52	0.01	1.30	2.47
11.89_412.2506m/z	N-oleoyl taurine	Fatty Acyls	4.46	0.00	3.46	10.98
13.08_387.2800n	N-palmitoyl methionine	Fatty Acyls	4.34	0.00	2.93	7.61
10.74_524.2791m/z	LysoPE(0:0/22:6(4Z,7Z,10Z,13Z,16Z,19Z))	Glycerophospholipids	4.33	0.02	2.91	7.52
2.28_603.2614m/z	PHOOA-PA	Glycerophospholipids	4.13	0.00	1.85	3.60

10.93_361.2729m/z	MG(0:0/20:4(5Z,8Z,11Z,14Z)/0:0)	Glycerolipids	4.11	0.01	2.66	6.33
11.96_338.2686m/z	20-hydroxy-eicosatetraenoic acid	Fatty Acyls	4.08	0.00	2.26	4.78
4.66_246.1698m/z	2-Methylbutyroylcarnitine	Fatty Acyls	3.99	0.00	1.87	3.66
12.45_302.2246n	EPA	Fatty Acyls	3.98	0.01	1.84	3.59
0.88_116.0706m/z	beta-vinyl acrylic acid	Fatty Acyls	3.96	0.02	0.23	1.17
1.21_360.1637n	Brosimacutin I	Polyketides	3.92	0.00	2.19	4.57
0.90_620.1553n	Apigenin 7-(3''-acetyl-6''-E-p-coumaroylglucoside)	Polyketides	3.89	0.00	-7.28	0.01
10.64_337.2735m/z	PGF2alpha methyl ether	Fatty Acyls	3.84	0.04	1.14	2.21
10.60_439.3635m/z	N-stearoyl histidine	Fatty Acyls	3.63	0.00	2.96	7.78
10.54_374.2821n	15-hydroxy-tetracosa-6,9,12,16,18-pentaenoic acid	Fatty Acyls	3.58	0.03	-0.87	0.55
1.76_358.9927m/z	Erosnin	Polyketides	3.52	0.00	-1.62	0.32
13.19_329.2493m/z	4,8,12,15,19-docosapentaenoic acid	Fatty Acyls	3.46	0.02	3.95	15.47
0.89_539.1374m/z	7,8,4'-Trihydroxy-3',5'-dimethoxyflavanone 4'-O-glucoside	Polyketides	3.45	0.00	-8.41	0.00

11.21_529.3160n	PE(22:4(7Z,10Z,13Z,16Z)/0:0)	Glycerophospholipids	3.44	0.03	1.19	2.27
3.75_437.2134m/z	PA(20:5(5Z,8Z,11Z,14Z,17Z)/0:0)	Glycerophospholipids	3.41	0.00	1.00	2.00
12.16_648.3259n	PI(22:4(7Z,10Z,13Z,16Z)/0:0)	Glycerophospholipids	3.22	0.02	4.07	16.79
0.86_173.0570m/z	2-amino-4-cyano-butanoic acid	Fatty Acyls	3.18	0.00	3.53	11.59
11.95_362.2685m/z	21-HDHA	Fatty Acyls	3.18	0.00	3.01	8.04
0.90_384.1258n	Gancaonin D	Polyketides	3.18	0.00	-4.12	0.06
0.93_142.0985n	4-ene-Valproic acid	Fatty Acyls	3.17	0.01	2.17	4.51
12.63_340.2867m/z	(-)-2,7-Dolabelladiene-6beta,10alpha,18-triol	Prenol lipids	3.16	0.01	1.93	3.81
11.38_572.3703m/z	LysoPC(22:4(7Z,10Z,13Z,16Z))	Glycerophospholipids	3.15	0.01	1.80	3.47
11.33_530.3230m/z	PE(20:1(11Z)/0:0)	Glycerophospholipids	3.12	0.01	1.62	3.06
4.71_565.6038m/z	Behenyl palmitate	Fatty Acyls	3.11	0.00	-2.23	0.21
0.84_379.0823m/z	Tephcalostan C	Polyketides	3.10	0.00	-4.60	0.04
11.76_647.3217m/z	OKOHA-PG	Glycerophospholipids	3.06	0.03	2.98	7.91
2.19_406.1216n	Viscutin 3	Polyketides	3.01	0.00	-3.33	0.10
11.63_508.2788n	PG(18:2(9Z,12Z)/0:0)	Glycerophospholipids	2.98	0.00	1.93	3.81
2.07_775.2115m/z	Sagittain B	Polyketides	2.97	0.00	-2.80	0.14

3.84_546.1668m/z	Mahuannin D	Polyketides	2.96	0.00	-10.55	0.00
11.29_507.2738m/z	1-(11Z,14Z-eicosadienoyl)-glycero-3-phosphate	Glycerophospholipids	2.94	0.05	1.85	3.60
10.31_461.3477m/z	(5Z,9Z)-2-methoxy-hexacosadienoic acid	Fatty Acyls	2.87	0.00	4.62	24.52
12.47_446.3255m/z	N-oleoyl tyrosine	Fatty Acyls	2.81	0.01	2.72	6.61
11.83_388.2960n	1alpha,25-dihydroxy-26,27-dinorvitamin D3	Sterol Lipids	2.80	0.02	2.91	7.54
13.03_361.1664m/z	(1E,4Z,6a,8b,10a)-8-(2-Methylbutanoyloxy)-10,15-dihydroxy-3-oxo-1,4,11(13)-germacratrien-12,6-olide	Prenol lipids	2.79	0.00	1.06	2.08
12.77_380.3154m/z	N-linoleoyl valine	Fatty Acyls	2.76	0.00	2.90	7.45
13.80_394.3327m/z	N-oleoyl isoleucine	Fatty Acyls	2.74	0.04	4.05	16.53
13.31_414.3027m/z	N-oleoyl methionine	Fatty Acyls	2.73	0.00	2.46	5.51
14.75_319.2629m/z	19:1(16Z)	Fatty Acyls	2.68	0.00	2.00	4.00
0.88_447.1345m/z	Hesperetin 7-O-rhamnoside	Polyketides	2.67	0.00	-7.34	0.01

12.21_352.2841m/z	21-hydroxyallopregnanolone	Sterol Lipids	2.61	0.00	2.73	6.62
13.45_330.2559n	4,7,10,13,16-docosapentaenoic acid	Fatty Acyls	2.59	0.04	1.38	2.60
1.18_160.1324m/z	(3Z)-2-Propylpent-3-enoic acid	Fatty Acyls	2.58	0.02	1.49	2.81
2.13_742.2009m/z	Platanoside	Polyketides	2.57	0.00	-2.59	0.17
14.98_807.5644n	PC(18:0/20:5(9Z,11Z,13Z,15Z,17Z))	Glycerophospholipids	2.57	0.02	1.66	3.16
0.91_763.1854m/z	Pelargonidin 3-(6''-p-coumarylglucoside)-5-(6'''-acetylglucoside)	Polyketides	2.57	0.00	-7.48	0.01
3.93_232.1541m/z	Isobutyryl-L-carnitine	Fatty Acyls	2.52	0.00	1.64	3.12
11.34_337.2732m/z	Pregnanetriol	Steroids and steroid derivatives	2.52	0.00	1.15	2.22
12.65_328.2841m/z	N-palmitoyl alanine	Fatty Acyls	2.51	0.00	2.70	6.50
13.03_269.2264m/z	9-cis-retinol	Prenol lipids	2.48	0.00	1.74	3.34
11.03_348.3023n	(E,E)-3,7,11-Trimethyl-2,6,10-dodecatrienyl octanoate	Fatty Acyls	2.47	0.01	2.29	4.89
10.20_409.3416m/z	24-vinyloxy-cholest-5,23Z-dien-3beta-ol	Sterol Lipids	2.46	0.00	3.71	13.09
9.31_318.2997m/z	12R-hydroxy-octadecanoic acid	Fatty Acyls	2.44	0.03	1.12	2.17

0.91_636.1831m/z	Malvidin 3-laminaribioside	Polyketides	2.44	0.00	-7.43	0.01
10.32_313.2387m/z	9,10-DiHOME	Fatty Acyls	2.42	0.00	-2.07	0.24
11.99_324.2897m/z	Bishomo-alpha-linolenic acid	Fatty Acyls	2.39	0.00	2.23	4.70
0.81_236.1728n	DB-2073	Polyketides	2.35	0.02	1.08	2.12
0.80_279.1906m/z	10-Deoxymethynolide	Polyketides	2.34	0.00	1.78	3.44
6.80_496.2730m/z	Tauro-b-muricholic acid	Steroids and steroid derivatives	2.33	0.02	2.04	4.12
10.65_546.2817m/z	PS(20:4(5Z,8Z,11Z,14Z)/0:0)	Glycerophospholipids	2.33	0.01	0.94	1.91
10.32_314.2453n	12,13-DiHOME	Fatty Acyls	2.29	0.00	-1.88	0.27
0.84_509.0898m/z	Isoaffinetin	Polyketides	2.28	0.00	-7.73	0.00
1.19_486.1694n	Gemichalcone B	Polyketides	2.26	0.00	-3.92	0.07
4.56_478.2369m/z	PE(15:0/0:0)	Glycerophospholipids	2.25	0.02	-0.98	0.51
0.83_162.0514n	2,3-dimethylmalic acid	Fatty Acyls	2.25	0.00	-3.42	0.09
3.75_475.1129n	Pelargonidin 3-(6''-acetylglucoside)	Polyketides	2.23	0.00	1.04	2.05
0.83_537.1652m/z	Okanin 3,4,3'-trimethyl ether 4'-glucoside	Polyketides	2.22	0.00	-7.92	0.00
9.70_288.2892m/z	10-methyl-hexadecanoic acid	Fatty Acyls	2.20	0.01	1.40	2.63

2.07_773.1971m/z	Viscumneoside V	Polyketides	2.20	0.00	-2.47	0.18
4.56_538.5310m/z	Cer(d18:0/16:0(2OH))	Sphingolipids	2.17	0.01	-1.69	0.31
11.25_409.3048m/z	N-(9,12-octadecadienoyl)-glutamine	Fatty Acyls	2.16	0.00	3.15	8.88
11.58_368.2788m/z	Tetrahydrodeoxycortisol	Sterol Lipids	2.15	0.00	2.67	6.38
4.95_599.3188m/z	PI(18:0/0:0)	Glycerophospholipids	2.15	0.05	2.34	5.05
13.03_509.2882m/z	PA(20:1(11Z)/0:0)	Glycerophospholipids	2.15	0.03	3.44	10.85
13.04_804.5504m/z	PC(22:4(7Z,10Z,13Z,16Z)/14:0)	Glycerophospholipids	2.14	0.02	1.04	2.06
0.85_541.1413m/z	Phellodensin F	Polyketides	2.14	0.00	-46.96	0.00
2.67_499.1146m/z	Oxytetracycline	Polyketides	2.14	0.00	-1.54	0.34
13.59_368.3173m/z	N-palmitoyl isoleucine	Fatty Acyls	2.14	0.02	2.80	6.96
13.04_220.1464n	Tetradecan-7,9-diynoic acid	Fatty Acyls	2.10	0.00	1.82	3.53
13.04_234.1612n	Macrophyllic acid A	Prenol lipids	2.10	0.00	1.70	3.24
13.62_390.2669m/z	(5Z,8Z)-tetradecadienoylcarnitine	Fatty Acyls	2.10	0.02	1.92	3.77
0.69_223.0243m/z	4-fumarylacetoacetic acid	Fatty Acyls	2.08	0.03	0.70	1.63
13.79_256.2406n	Palmitic acid	Fatty Acyls	2.07	0.01	1.21	2.32
0.81_309.1287m/z	estra-1,3,5(10),7-tetraene-3,17alpha-diol	Sterol Lipids	2.06	0.03	-0.78	0.58

10.93_585.2805m/z	PKOOA-PA	Glycerophospholipids	2.04	0.01	2.84	7.15
10.54_392.2925n	Ursodeoxycholic acid	Steroids and steroid derivatives	2.04	0.02	-1.01	0.50
9.65_787.4196m/z	Asprellic acid A	Prenol lipids	2.03	0.02	2.78	6.86
2.09_241.1543m/z	Cerulenin	Fatty Acyls	2.03	0.02	0.40	1.32
10.31_485.3474m/z	(25S)-5alpha-cholestan-3beta,4beta,6alpha,8beta,15alpha,16beta,26-heptol	Sterol Lipids	2.03	0.00	5.01	32.23
12.81_368.3153m/z	13-(3,4-dimethyl-5-propylfuran-2-yl)-tridecanoic acid	Fatty Acyls	2.02	0.00	2.54	5.80
13.82_331.2647m/z	Adrenic Acid	Fatty Acyls	2.01	0.04	2.15	4.42
1.99_487.1304m/z	Mikanin 3-galactoside	Polyketides	2.01	0.00	1.66	3.17
13.10_510.2961n	PG(18:1(9Z)/0:0)	Glycerophospholipids	2.01	0.04	4.65	25.03
8.45_391.2833m/z	Octadecyl fumarate	Fatty Acyls	2.00	0.02	1.65	3.13
0.85_738.1510m/z	Malvidin 3-glucoside-5-(6-acetylglucoside)	Polyketides	2.00	0.00	-46.76	0.00
13.81_610.4068n	6-O-(Glcb)-(25R)-5alpha-spirostan-3beta,6alpha-diol	Sterol Lipids	2.00	0.01	-2.76	0.15

10.65_385.2730m/z	1alpha-hydroxy-26,27-dinorvitamin D3 25-carboxylic acid	Sterol Lipids	1.99	0.01	2.19	4.57
10.46_422.3255m/z	Palmitoylcarnitine	Fatty Acyls	1.99	0.00	3.65	12.53
11.38_328.2607n	Avocadene 2-acetate	Fatty Acyls	1.99	0.02	-1.44	0.37
10.88_337.2731m/z	10-methoxy-octadecanoic acid	Fatty Acyls	1.97	0.03	1.65	3.14
15.75_271.2621m/z	Isopropyl tetradecanoate	Fatty Acyls	1.96	0.00	0.54	1.45
10.91_627.2914m/z	PI(22:6(4Z,7Z,10Z,13Z,16Z,19Z)/0:0)	Glycerophospholipids	1.96	0.01	3.70	12.97
0.86_547.1979m/z	Tomentosanol E	Polyketides	1.95	0.00	-2.79	0.14
0.75_215.0550n	sn-glycero-3-phosphoethanolamine	Glycerophospholipids	1.95	0.00	-1.53	0.35
11.34_508.2786n	26,26,26,27,27,27-hexafluoro-25-hydroxyvitamin D3	Sterol Lipids	1.94	0.00	1.46	2.74
10.72_298.2504n	12R-HOME(13E)	Fatty Acyls	1.94	0.01	-2.70	0.15
2.13_740.1866m/z	Delphinidin 3-(2-xylosylgalactoside)-5-glucoside	Polyketides	1.94	0.00	-3.02	0.12
10.36_411.3085n	N-oleoyl glutamic acid	Fatty Acyls	1.94	0.00	2.03	4.08
1.13_665.2138m/z	Matterionate A	Polyketides	1.93	0.00	-10.84	0.00

2.02_538.1262m/z	5,3',4'-Trihydroxy-3-methoxy-6,7-methylenedioxyflavone 4'-glucuronide	Polyketides	1.92	0.00	-2.50	0.18
13.50_382.3309m/z	13-(3-methyl-5-pentylfuran-2-yl)-tridecanoic acid	Fatty Acyls	1.88	0.00	2.69	6.47
0.91_591.1771m/z	Baicalein 6-methyl ether 7-glucosyl-(1->3)-rhamnoside	Polyketides	1.87	0.00	-4.11	0.06
10.53_411.3570m/z	1alpha-hydroxy-26,27-dimethylvitamin D3	Sterol Lipids	1.86	0.00	3.11	8.65
0.91_703.1740m/z	6'''-(3-Hydroxy-3-methylglutaroyl)isoviolanthin	Polyketides	1.86	0.00	-12.44	0.00
2.16_448.2090m/z	PC(5:0/5:0)	Glycerophospholipids	1.85	0.01	0.50	1.41
10.72_315.2542m/z	9R,10S-dihydroxy-stearic acid	Fatty Acyls	1.85	0.00	-2.62	0.16
10.86_450.3569m/z	(23S)-23,24,25-trihydroxyvitamin D3	Sterol Lipids	1.85	0.01	2.88	7.38
11.03_476.3720m/z	18-acetoxy-25-hydroxyvitamin D3	Sterol Lipids	1.85	0.01	6.33	80.65
8.69_530.2519m/z	Epothilone B	Polyketides	1.84	0.00	6.22	74.33

10.50_316.3206m/z	5-methyl-octadecanoic acid	Fatty Acyls	1.79	0.03	1.29	2.45
11.80_492.3102m/z	PC(14:0/2:0)	Glycerophospholipids	1.78	0.00	4.25	18.99
0.77_399.1422m/z	Hoslunddiol	Polyketides	1.78	0.00	-2.36	0.19
11.82_382.2944m/z	11-deoxy-16,16-dimethyl-PGE2	Fatty Acyls	1.76	0.00	3.33	10.07
11.13_526.2942m/z	PE(22:5(4Z,7Z,10Z,13Z,16Z)/0:0)	Glycerophospholipids	1.76	0.03	1.92	3.77
10.98_279.2312m/z	13(S)-HODE	Fatty Acyls	1.75	0.00	-2.19	0.22
10.82_529.3107m/z	26,27-diethyl-1alpha,25-dihydroxy-22-thia-20-epivitamin D3	Sterol Lipids	1.73	0.00	1.83	3.56
10.93_675.2203m/z	Scutellarein 6,7,4'-trimethyl ether 5-(6'''-acetylglucosyl)(1->3)-galactoside	Polyketides	1.71	0.00	1.90	3.74
4.56_431.2274m/z	L-Linalool 3-[xylosyl-(1->6)-glucoside]	Fatty Acyls	1.70	0.01	-1.50	0.35
12.21_358.2947m/z	N-palmitoyl threonine	Fatty Acyls	1.70	0.00	3.22	9.32
12.96_452.3146m/z	N-oleoyl phenylalanine	Fatty Acyls	1.70	0.00	10.39	1342.70
13.18_224.1772n	14:2(6,9)	Fatty Acyls	1.70	0.00	0.96	1.94

12.85_293.2289m/z	11Z-heptadecen-1-ol	Fatty Acyls	1.69	0.00	3.46	11.01
0.76_249.0839n	S-Acetyldihydrolipoamide	Fatty Acyls	1.69	0.00	-1.06	0.48
11.24_297.2438m/z	Isoricinoleic Acid	Fatty Acyls	1.68	0.01	-1.43	0.37
9.49_230.2474m/z	Xestoaminol C	Sphingolipids	1.67	0.05	0.87	1.83
10.49_442.3517m/z	(9Z)-3-hydroxyoctadecenoylcarnitine	Fatty Acyls	1.64	0.05	3.23	9.37
4.56_637.6469m/z	Hydroxyphthioceranic acid (C42)	Fatty Acyls	1.64	0.01	-1.08	0.47
9.66_944.5014m/z	Tragopogonsaponin B	Prenol lipids	1.64	0.03	2.40	5.28
9.84_311.2224m/z	8R-HpODE	Fatty Acyls	1.63	0.00	-2.88	0.14
4.23_360.0845m/z	Rosinidin	Polyketides	1.62	0.00	-1.61	0.33
11.80_411.3218m/z	N-oleoyl glutamine	Fatty Acyls	1.62	0.00	2.71	6.56
10.94_441.3786m/z	30:6(12Z,15Z,18Z,21Z,24Z,27Z)	Fatty Acyls	1.62	0.00	3.08	8.47
4.06_601.1989m/z	Loganin pentaacetate	Prenol lipids	1.62	0.00	2.52	5.73
0.83_519.2016m/z	11-Oxo-androsterone glucuronide	Sterol Lipids	1.61	0.00	-6.72	0.01
0.68_436.1091n	9,10-Dihydro-10-(4-hydroxyphenyl)-pyrano[2,3-h]epicatechin-8-one	Polyketides	1.61	0.05	0.93	1.90

10.25_302.3045m/z	Enigmol	Sphingolipids	1.61	0.04	1.25	2.38
0.84_719.2013m/z	Apigenin 7-rhamnosyl-(1->6)-(4''-E-p-methoxycinnamoylglucoside)	Polyketides	1.60	0.00	-6.11	0.01
2.07_596.1328m/z	Delphinidin 3-sambubioside	Polyketides	1.59	0.00	0.57	1.48
8.80_482.2935m/z	Taurolithocholic acid	Sterol Lipids	1.59	0.00	3.72	13.17
2.03_917.2211m/z	Kaempferol 3-(6'''-rhamnosyl-2'''-(6-malyl-glucosyl)-glucoside)	Polyketides	1.57	0.00	-4.35	0.05
2.06_103.0398m/z	D(-)-beta-hydroxy butyric acid	Fatty Acyls	1.57	0.00	1.59	3.01
11.99_348.2902m/z	22-hydroxy-23,24,25,26,27-pentanorvitamin D3	Sterol Lipids	1.57	0.00	5.70	52.02
14.41_343.2637m/z	1alpha,23-dihydroxy-24,25,26,27-tetranorvitamin D3	Sterol Lipids	1.54	0.00	2.41	5.31
1.46_229.1538m/z	11Z-Hexadecen-7,9-diynoic acid	Fatty Acyls	1.54	0.00	1.29	2.44
11.42_524.2972m/z	PG(18:3(9Z,12Z,15Z)/0:0)	Glycerophospholipids	1.53	0.04	1.17	2.24

10.95_390.3362m/z	(+)-Dysideapalaunic acid	Prenol lipids	1.53	0.00	10.29	1248.94
0.80_405.1173m/z	Chalconaringenin 2'-xyloside	Polyketides	1.53	0.00	1.49	2.80
13.03_363.1620m/z	Ecabet	Prenol lipids	1.52	0.00	1.01	2.02
11.15_362.2351m/z	Formyldienolone	Sterol Lipids	1.52	0.00	2.15	4.44
2.04_598.1465m/z	Delphinidin 3-lathyroside	Polyketides	1.52	0.00	1.66	3.16
2.07_436.1256n	Epigallocatechin 3-O-cinnamate	Polyketides	1.51	0.03	-2.26	0.21
2.24_448.1434n	Poriolin	Polyketides	1.51	0.00	-7.31	0.01
10.96_590.3471m/z	PC(20:3(5Z,8Z,11Z)/0:0)	Glycerophospholipids	1.50	0.05	-1.26	0.42
1.17_337.0753m/z	Isognaphalin 8-acetate	Polyketides	1.50	0.00	-1.78	0.29
0.91_254.0996n	Galactosylglycerol	Glycerolipids	1.50	0.00	-2.54	0.17
2.16_397.2549m/z	PGF2alpha-11-acetate	Fatty Acyls	1.50	0.00	-3.95	0.06
10.78_200.2004m/z	dodecanamide	Fatty Acyls	1.50	0.01	0.71	1.63
1.23_442.1908m/z	18-Carboxy-dinor-LTE4	Fatty Acyls	1.49	0.00	-7.75	0.00
12.26_252.2085n	8Z,10Z-hexadecadienoic acid	Fatty Acyls	1.47	0.00	2.46	5.49
13.74_430.3309m/z	Episceptrumgenin	Sterol Lipids	1.47	0.01	3.37	10.33
10.78_474.3573m/z	(22S)-1alpha,22,25-trihydroxy-26,27-dimethyl-	Sterol Lipids	1.47	0.02	7.37	165.71

	23,23,24,24-tetradehydro vitamin D3					
12.33_304.2630m/z	Arachidonoyl amine	Fatty Acyls	1.47	0.00	46.03	71768034358585.70
12.24_418.2964m/z	N-palmitoyl tyrosine	Fatty Acyls	1.46	0.03	3.03	8.15
1.51_633.1807m/z	Hesperidin	Polyketides	1.45	0.00	6.14	70.69
12.44_301.2178m/z	Eicosapentaenoic Acid	Fatty Acyls	1.44	0.02	1.89	3.70
1.77_291.1808m/z	(R)-1-O-b-D-glucopyranosyl-1,3-octanediol	Fatty Acyls	1.44	0.00	3.85	14.46
10.06_411.3320m/z	N-palmitoyl histidine	Fatty Acyls	1.43	0.00	3.02	8.10
12.85_387.1614m/z	Puerarol	Polyketides	1.42	0.00	2.28	4.85
0.85_712.7009m/z	20:0 Campesterol ester	Sterol Lipids	1.42	0.00	-45.82	0.00
1.53_631.1653m/z	6-Methoxykaempferol 3-rhamnoside-7-(4'''-acetylrhamnoside)	Polyketides	1.42	0.00	4.50	22.68
11.38_368.3515m/z	20:2(5Z,9Z)(11Me,15Me,19Me)	Prenol lipids	1.41	0.00	8.26	307.28
0.76_523.1035m/z	Syringetin 3-glucuronide	Polyketides	1.41	0.00	1.32	2.49
0.76_381.1079m/z	Torosaflavone A	Polyketides	1.41	0.00	-0.62	0.65
0.84_903.2706m/z	Epimedokoreanoside I	Polyketides	1.41	0.00	-4.04	0.06

0.86_349.1345m/z	2',6'-Dihydroxy-4'-prenyloxydihydrochalcone	Polyketides	1.40	0.00	6.42	85.87
7.42_543.3446m/z	Cucurbitacin Q	Sterol Lipids	1.40	0.03	1.19	2.28
0.90_624.2336m/z	Embinin	Polyketides	1.40	0.00	-45.75	0.00
0.92_647.1088m/z	6-Hydroxyluteolin 7-[3''-(3-hydroxy-3-methylglutaryl)glucoside]	Polyketides	1.40	0.00	45.93	66928735915764.20
0.90_612.1759n	Eriodictyol 5,3'-di-O-glucoside	Polyketides	1.40	0.00	-7.62	0.01
3.92_454.2313m/z	Orotinichalcone	Polyketides	1.40	0.00	-2.30	0.20
2.02_374.1298m/z	Isokanugin	Polyketides	1.40	0.01	3.07	8.40
2.17_527.1096m/z	Apigenin 7-(2''-glucosyllactate)	Polyketides	1.39	0.01	2.68	6.40
11.42_626.4014n	6-O-(Glcb)-(25R)-5alpha-spirostan-3beta,6alpha,23S-triol	Sterol Lipids	1.39	0.00	-5.10	0.03
11.87_466.2964m/z	N-arachidonoyl tyrosine	Fatty Acyls	1.38	0.01	4.43	21.51
10.24_433.3409m/z	Delta 8,14 -Sterol	Sterol Lipids	1.38	0.00	7.38	166.25
10.29_435.3335m/z	Masticadienonic acid	Sterol Lipids	1.38	0.02	4.57	23.82
0.85_837.1309m/z	Luteolin 7,3',4'-triglucuronide	Polyketides	1.37	0.00	-45.69	0.00

11.36_280.2400n	10E,12Z-Octadecadienoic acid	Fatty Acyls	1.37	0.01	-1.00	0.50
13.04_168.1894n	1-Dodecene	Fatty Acyls	1.37	0.01	2.06	4.16
10.05_370.2944m/z	2-Hydroxymyristoylcarnitine	Fatty Acyls	1.36	0.02	2.02	4.04
4.56_717.7051m/z	24:1 Cholesterol ester	Sterol Lipids	1.36	0.01	-1.83	0.28
2.37_325.1069m/z	6-hydroxy-7E,9E-Octadecadiene-11,13,15,17-tetraynoic acid	Fatty Acyls	1.36	0.00	1.19	2.28
14.24_310.3099m/z	3,7,11,15-Tetramethyl-6,10,14-hexadecatrien-1-ol	Fatty Acyls	1.35	0.03	0.93	1.91
0.92_798.2309m/z	Malvidin 3-gentiotrioside	Polyketides	1.35	0.00	-7.53	0.01
10.12_842.6619m/z	PC(16:1(9Z)/24:1(15Z))	Glycerophospholipids	1.34	0.00	1.70	3.24
0.86_515.1719m/z	Gemichalcone A	Polyketides	1.34	0.00	-2.95	0.13
1.52_473.0946m/z	Isocarthamidin 7-O-rhamnoside	Polyketides	1.34	0.00	3.24	9.45
11.63_473.2648m/z	PS(13:0/0:0)	Glycerophospholipids	1.33	0.00	1.89	3.72
14.15_416.3185m/z	25,26-epoxy-1alpha-hydroxy-23,23,24,24-tetradehydro-19-norvitamin D3	Sterol Lipids	1.33	0.00	3.09	8.53

9.65_787.7539m/z	34-carboxy-(tritriacont-9Z-enyl) oleate	Fatty Acyls	1.33	0.02	3.43	10.81
0.92_753.2316m/z	Diosmetin 7-(2'',6''-dirhamnosyl)-glucoside	Polyketides	1.33	0.00	-3.35	0.10
10.97_614.3470m/z	PC(22:5(7Z,10Z,13Z,16Z,19Z)/0:0)	Glycerophospholipids	1.32	0.02	3.73	13.27
2.29_749.2218m/z	Isoliquiritigenin 4-O-(5'''-O-feruloyl)-apiofuranosyl-(1'''->2'')-glucoside	Polyketides	1.32	0.01	46.00	70129792671484.00
0.92_607.1715m/z	Dalmaisione D	Polyketides	1.32	0.00	-12.66	0.00
1.19_830.1309m/z	2-methylcrotonoyl-CoA	Fatty Acyls	1.31	0.00	2.04	4.10
12.61_461.1962m/z	Kuraridin	Polyketides	1.31	0.00	3.40	10.52
6.80_497.2796n	1-(2-methoxy-6Z-pentadecenyl)-sn-glycero-3-phosphoserine	Glycerophospholipids	1.30	0.02	1.85	3.60
2.29_391.1086m/z	Thonningine B	Polyketides	1.30	0.00	3.94	15.33
2.11_237.0071m/z	(E)-4-(6-methyl-1,2-dithiin-3-yl)but-1-en-3-yn-1-yl acetate	Fatty Acyls	1.30	0.03	0.80	1.74
12.20_370.2945m/z	cis-5-Tetradecenoylcarnitine	Fatty Acyls	1.30	0.00	2.16	4.46

13.18_313.2732m/z	3-oxo-nonadecanoic acid	Fatty Acyls	1.29	0.03	0.65	1.57
4.56_431.4275m/z	4-methyl-stigmastan-3beta-ol	Sterol Lipids	1.29	0.01	-1.51	0.35
12.57_434.2734m/z	Sphingofungin B	Sphingolipids	1.29	0.01	3.99	15.91
1.92_262.1283m/z	Methylmalonylcarnitine	Fatty Acyls	1.29	0.00	-3.69	0.08
10.86_614.3466m/z	OHOOA-PE	Glycerophospholipids	1.28	0.03	1.75	3.36
3.88_407.2221m/z	15-methyl-15S-PGE1	Fatty Acyls	1.28	0.00	-1.44	0.37
15.04_295.2626m/z	1,17-Heptadecanediol	Fatty Acyls	1.27	0.02	1.09	2.13
4.46_457.1107m/z	Floribundoside	Polyketides	1.27	0.02	0.77	1.71
0.73_333.0579m/z	1-(sn-Glycero-3-phospho)-1D-myo-inositol	Glycerophospholipids	1.27	0.00	0.83	1.78
12.49_392.3150m/z	3alpha-Hydroxy-5beta-chol-7-en-24-oic Acid	Sterol Lipids	1.27	0.00	2.59	6.01
11.29_433.3049m/z	calicoferol D	Sterol Lipids	1.27	0.00	5.86	57.92
11.43_454.3880m/z	3-hydroxyeicosanoylcarnitine	Fatty Acyls	1.26	0.00	2.72	6.60
10.15_440.3361m/z	3-hydroxylinoleoylcarnitine	Fatty Acyls	1.26	0.03	3.31	9.94
0.90_580.2076m/z	Sophoraflavone A	Polyketides	1.26	0.00	-45.53	0.00

0.89_707.2341m/z	Ikarisoside B	Polyketides	1.25	0.00	-4.44	0.05
0.89_426.1317n	Dalbinol	Polyketides	1.25	0.00	-4.95	0.03
13.03_403.1580m/z	Albanin E	Polyketides	1.25	0.01	3.58	11.96
10.71_560.2972m/z	PS(19:1(9Z)/0:0)	Glycerophospholipids	1.24	0.01	3.82	14.08
10.91_271.2284m/z	9-methoxy-pentadecanoic acid	Fatty Acyls	1.24	0.02	-1.10	0.47
0.77_294.0812m/z	(S)-malyl alpha-D-glucosaminide	Fatty Acyls	1.22	0.00	-1.55	0.34
2.49_370.1374n	Dehydrocycloxanthohumol hydrate	Polyketides	1.22	0.00	-2.05	0.24
11.67_312.2528m/z	2-HoTrE	Fatty Acyls	1.21	0.00	1.88	3.68
0.76_132.0645m/z	2-amino-4-oxo-pentanoic acid	Fatty Acyls	1.21	0.01	-0.51	0.70
11.55_574.3118m/z	PS(20:1(11Z)/0:0)	Glycerophospholipids	1.21	0.00	1.88	3.68
11.62_418.2943m/z	(7Z,10Z)-hexadecadienoylcarnitine	Fatty Acyls	1.20	0.01	3.03	8.18
11.61_392.2788m/z	15-HETE-Ala	Fatty Acyls	1.20	0.00	3.85	14.37
0.90_948.2669n	Isorhamnetin 3-rhamnosyl-(1->2)-gentiobiosyl-(1->6)-glucoside	Polyketides	1.19	0.00	-5.61	0.02
2.56_625.1593m/z	4,2',3',4'-Tetrahydroxy	Polyketides	1.19	0.00	-3.08	0.12

0.92_769.2246m/z	chalcone 4'-O-(2''-O-p-coumaroyl)glucoside 4'-Hydroxy-5,7-dimethoxyflavanone 4'-[2-(5-Cinnamoyl)-beta-D-apiofuranosyl]glucoside	Polyketides	1.19	0.00	-7.79	0.00
2.73_497.0998m/z	Apigenin 7-(2'',3''-diacetylglucoside)	Polyketides	1.19	0.00	-1.47	0.36
0.83_450.1259m/z	17-Beta-Estradiol-3,17-beta-sulfate	Sterol Lipids	1.19	0.00	-45.32	0.00
4.25_563.2167m/z	Leucadenone B	Polyketides	1.19	0.00	-4.21	0.05
2.03_452.1867m/z	Neoraufuracin	Polyketides	1.18	0.04	2.36	5.13
0.80_166.0524m/z	2-oxo-4-methylthio-butanoic acid	Fatty Acyls	1.18	0.01	0.65	1.57
12.75_402.2989m/z	N-oleoyl proline	Fatty Acyls	1.18	0.01	4.32	20.01
13.19_763.5958m/z	PC(18:1(9Z)/15:0)	Glycerophospholipids	1.17	0.02	0.70	1.62
10.80_559.2976m/z	Hippurin-1	Sterol Lipids	1.17	0.03	1.95	3.88
9.84_277.2154m/z	9-OxoODE	Fatty Acyls	1.16	0.00	-2.28	0.21
4.31_698.3844m/z	PS(14:1(9Z)/14:1(9Z))	Glycerophospholipids	1.16	0.00	-2.49	0.18
0.78_211.0820m/z	1-Deoxy-D-glucitol	Fatty Acyls	1.16	0.02	1.70	3.25

12.84_283.2437m/z	18-fluoro-octadecanoic acid	Fatty Acyls	1.16	0.02	2.54	5.82
10.31_416.3360m/z	3-hydroxyhexadecanoyl carnitine	Fatty Acyls	1.15	0.05	3.25	9.50
11.63_337.2730m/z	2-hydroxy-nonadecanoic acid	Fatty Acyls	1.15	0.01	0.81	1.76
11.83_631.3227m/z	OOV-PG	Glycerophospholipids	1.15	0.03	3.81	13.99
11.77_442.1691m/z	Ginkgolide J	Prenol lipids	1.14	0.00	2.84	7.18
10.42_446.3256m/z	(23S,25R)-25-hydroxyvitamin D3 26,23-lactone	Sterol Lipids	1.14	0.00	4.64	24.98
9.64_368.2788m/z	11-deoxy-11-methylene-PGD2	Fatty Acyls	1.13	0.00	2.83	7.09
0.74_558.0209n	Quercetin 3-glucuronide-7-sulfate	Polyketides	1.13	0.01	0.73	1.66
10.62_333.2069m/z	20-hydroxy LTB4	Fatty Acyls	1.13	0.02	1.18	2.27
9.26_318.2809m/z	18-fluoro-9Z-octadecenoic acid	Fatty Acyls	1.13	0.03	0.99	1.99
0.91_161.0684n	Aminoadipic acid	Fatty Acyls	1.13	0.00	2.13	4.38
2.52_285.0826m/z	3,4,3',4'-Tetrahydroxy-2-methoxychalcone	Polyketides	1.12	0.01	-0.77	0.58
12.83_476.3153m/z	N-docosahexaenoyl	Fatty Acyls	1.12	0.03	45.81	61544791111388.20

	phenylalanine					
13.60_328.3205m/z	N,N-dimethylsphingosine	Sphingolipids	1.12	0.04	0.97	1.96
4.73_588.3083n	OKOOA-PA	Glycerophospholipids	1.11	0.00	-1.35	0.39
2.03_917.7216m/z	PA(24:0/24:0)	Glycerophospholipids	1.11	0.00	-4.69	0.04
11.09_295.2282m/z	9R,10S-EpOME	Fatty Acyls	1.11	0.00	-1.20	0.44
12.16_389.3042m/z	1alpha,25-dihydroxy-19-nor-22-oxavitamin D3	Sterol Lipids	1.11	0.01	7.37	165.62
11.75_282.2553n	Oleic acid	Fatty Acyls	1.11	0.02	-1.06	0.48
14.90_345.2783m/z	24-Nor-5beta-chol-22-ene-3alpha,7alpha,12alpha-triol	Sterol Lipids	1.11	0.00	3.28	9.73
13.03_690.3964m/z	OHODiA-PC	Glycerophospholipids	1.10	0.00	4.01	16.12
11.40_387.2887m/z	1alpha,25-dihydroxy-21-nor-20-oxavitamin D3	Sterol Lipids	1.10	0.03	1.61	3.05
4.31_672.3662m/z	OON-PE	Glycerophospholipids	1.10	0.00	-3.68	0.08
12.21_350.2705m/z	O-(2-tetradecenoyl)carnitine	Fatty Acyls	1.10	0.01	2.96	7.80
2.13_434.1333m/z	Calomelanol D	Polyketides	1.09	0.03	11.07	2146.07
11.94_468.4029m/z	6-Deoxocastasterone	Sterol Lipids	1.09	0.00	3.28	9.73

0.74_481.0925m/z	Villol	Polyketides	1.09	0.00	1.57	2.98
0.80_395.1919m/z	Isopentyl gentiobioside	Fatty Acyls	1.08	0.04	1.91	3.76
10.68_482.3227m/z	LysoPC(15:0)	Glycerophospholipids	1.08	0.02	-2.10	0.23
3.53_290.1594m/z	3-Methylglutarylcarnitine	Fatty Acyls	1.08	0.02	3.01	8.07
9.20_633.2541m/z	Ichangin 4-glucoside	Prenol lipids	1.08	0.02	1.95	3.86
5.07_712.4060m/z	PS(17:2(9Z,12Z)/12:0)	Glycerophospholipids	1.08	0.00	-2.64	0.16
11.84_324.2891m/z	N-palmitoyl GABA	Fatty Acyls	1.07	0.03	1.81	3.50
1.98_205.0350m/z	3-oxo-adipic acid	Fatty Acyls	1.07	0.00	1.71	3.28
4.17_524.1516m/z	6-Methoxyluteolin 7-glucuronide methyl ester	Polyketides	1.07	0.00	-11.14	0.00
0.81_273.1116m/z	3',4'-Dihydroxy-7-methoxyflavan	Polyketides	1.07	0.02	-0.96	0.51
10.84_444.3674m/z	12-Hydroxy-12-octadecanoylcarnitine	Fatty Acyls	1.07	0.03	2.98	7.91
13.18_379.1580m/z	3beta,4beta,5-Trimethoxy-4'-hydroxy-(6:7)-2,2-dimethylpyranoflavan	Polyketides	1.06	0.04	1.80	3.48
1.77_305.0722m/z	Alphitonin	Polyketides	1.06	0.00	-2.65	0.16
12.61_428.3153m/z	1alpha,25-dihydroxy-16,17,23,23,	Sterol Lipids	1.06	0.01	2.66	6.33

	24,24-hexadehydrovitamin D3					
4.11_529.5266m/z	Cer(d18:0/14:0)	Sphingolipids	1.06	0.00	4.53	23.11
15.46_675.6757m/z	Archaeol	Glycerolipids	1.06	0.03	1.12	2.17
11.58_366.2654m/z	Anandamide (18:3, n-6)	Fatty Acyls	1.06	0.01	2.64	6.22
12.03_254.2477m/z	4E,6E,10Z-Hexadecatrien-1-ol	Fatty Acyls	1.06	0.01	2.18	4.53
0.89_931.2660m/z	Sempervirenoside A	Polyketides	1.05	0.00	-45.13	0.00
4.72_489.2545m/z	2beta-chloro-1alpha,25-dihydroxyvitamin D3	Sterol Lipids	1.05	0.02	-1.52	0.35
1.19_550.1672n	Liquiritigenin 7-apiofuranoside-4'-glucoside	Polyketides	1.04	0.00	-3.48	0.09
3.75_461.2093m/z	PA(17:1(9Z)/0:0)	Glycerophospholipids	1.04	0.01	1.27	2.41
4.17_501.2633m/z	2-dehydroecdysone	Sterol Lipids	1.04	0.01	-1.35	0.39
2.51_262.1644m/z	3-hydroxyisovalerylcarnitine	Fatty Acyls	1.04	0.01	0.54	1.45
5.64_970.2742m/z	Negretein	Polyketides	1.03	0.00	3.42	10.71
11.96_262.2296n	5-(2-methoxyethyl)isolongifol-5-ene	Prenol lipids	1.03	0.00	1.65	3.13
13.04_114.1428n	octane	Fatty Acyls	1.03	0.02	2.64	6.25
15.04_262.2297n	6,10,14-Trimethyl-5,9,13-	Prenol lipids	1.03	0.02	0.89	1.85

	pentadecatrien-2-one					
6.30_814.2047n	Luteorin 7-(6''''-acetylallosyl-(1->3)-glucosyl-(1->2)-glucoside	Polyketides	1.03	0.02	0.26	1.20
4.88_586.3085m/z	leukotriene F4	Fatty Acyls	1.03	0.00	-1.48	0.36
10.97_295.2278m/z	9(S)-HODE	Fatty Acyls	1.02	0.00	-2.86	0.14
0.87_306.1403m/z	4,2',4'-Trihydroxy-3-methoxydihydrochalcone	Polyketides	1.02	0.00	6.35	81.62
1.98_338.0742m/z	PC(2:0/0:0)	Glycerophospholipids	1.02	0.02	0.57	1.48
10.93_269.2257m/z	13-cis-retinol	Prenol lipids	1.02	0.02	2.23	4.70
10.37_618.2785n	PI(20:5(5Z,8Z,11Z,14Z,17Z)/0:0)	Glycerophospholipids	1.01	0.03	3.20	9.17
6.48_285.0754m/z	Galangin 3-methyl ether	Polyketides	1.01	0.02	-1.05	0.48
2.91_378.1319m/z	Pinocembrin 7-O-benzoate	Polyketides	1.01	0.03	3.34	10.14
7.26_269.0442m/z	Apigenin	Polyketides	1.01	0.00	-2.24	0.21
13.04_166.0976n	Carvone oxide	Prenol lipids	1.00	0.00	2.05	4.13
9.80_480.2777m/z	Tauroursodeoxycholic acid	Sterol Lipids	1.00	0.00	3.66	12.61
2.27_230.1384m/z	3-hydroxybutyrylcarnitine	Fatty Acyls	1.00	0.00	-2.77	0.15
